# Sticky Yet Slippery: Molecular Ordering Reconciles Bubble‐Surface Affinity With Ultralow Friction at the Nanoscale

**DOI:** 10.1002/advs.75495

**Published:** 2026-05-07

**Authors:** Shishuang Zhang, Jiajun Li, Huadong Tian, Zhoujie Wang, Chenyu Qiao, Jingyi Wang, Lei Xie, Hongbo Zeng

**Affiliations:** ^1^ School of Minerals Processing and Bioengineering Central South University Changsha P. R. China; ^2^ Future Resources Interface Science and Intelligent Application Research Center (FRISIARC) Changsha P. R. China; ^3^ Department of Chemical and Materials Engineering University of Alberta Edmonton Canada; ^4^ College of Chemistry and Chemical Engineering Southwest Petroleum University Chengdu P. R. China

**Keywords:** AFM force measurements, bubble transport, bubble‐surface interactions, friction forces, hydrophobic interactions, molecular order, soft interfaces

## Abstract

A general understanding in wetting is that droplet stickiness and slipperiness are independent and do not necessarily change synchronously. However, it remains unclear whether bubbles behave similarly, making it challenging to achieve both strong interfacial affinity and low‐friction mobility, crucial for controlling bubble dynamics in microfluidics, catalysis, and gas‐liquid separation. Here, we show that molecular ordering within surface‐tethered polydimethylsiloxane (PDMS) layers reconciles bubble stickiness and slipperiness, enabling bubbles to maintain robust attachment and ultralow friction. Remarkably, tuning PDMS from disordered networks to ordered brushes nearly doubles hydrophobic decay length (*D*_0_, ∼0.8 to ∼1.4 nm) while significantly reducing friction, as quantified by bubble probe atomic force microscopy. Increasing brush thickness further extends *D*_0_ to ∼1.9 nm and reduces friction by additional 60%. Molecular dynamics simulations attribute this “sticky‐slippery” synergy to molecular ordering and homogenization, which reorganize interfacial water molecules into a well‐defined depletion layer that strengthens hydrophobic interaction; simultaneously, coherent chain tilting and reorientation during bubble sliding preserve chain order and packing density, minimizing contact line pinning and energy dissipation. These findings unveil a universal molecular mechanism that reconciles bubble affinity with ultralow friction, establishing a general framework for engineering adaptive soft interfaces with programmable wetting, adhesion, and interfacial transport properties.

## Graphical abstract

By engineering the molecular order and thickness of PDMS layers, we reconcile the stickiness and slipperiness during bubble transport. AFM measurements and MD simulations further reveal how these nanoscale architectures tune hydrophobic interaction *F*_HB_ and friction force *f*. This work offers a general paradigm for designing adaptive soft surfaces tailored for bubble transport, catalysis, microfluidic, and other gas‐involved processes.

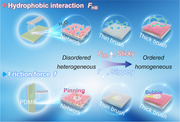

## 1 Introduction

The motion of water droplets on solid surfaces is ubiquitous in nature and daily life, where the interactions at the solid‐liquid‐gas interface play a crucial role in determining wetting, adhesion, and transport behaviors [1, 2, 3, 4]. As another important form of multiphase interfacial systems, air bubbles on solid surfaces in aqueous environments are indispensable for the survival of submerged organisms, and their movement is of great significance in a wide range of interfacial applications, including heterogeneous catalysis, electrochemical reactions, froth flotation, and microfluidics [5, 6, 7]. In the wetting field it is recognized that droplet adhesion and droplet friction are decoupled concepts: high adhesion does not necessarily correlate with high friction, and vice versa [8]. For example, low‐friction hydrophilic surfaces exhibit high adhesion, as reflected by low static contact angles, while still supporting facile droplet motion with low contact angle hysteresis [9, 10, 11, 12]. During sliding, droplet friction can be decomposed into two primary components: a normal force governed by surface tension and wettability, and a friction force associated with contact line dissipation [13, 14]. In contrast to water droplets in air, underwater bubbles possess a highly deformable gas‐liquid interface confined by the surrounding liquid, whose unique property like gas compressibility probably make bubble dynamics fundamentally different from the inverse of droplet wetting [15]. Such distinction raises a critical question: does the decoupling between adhesion and friction for droplets remain valid for underwater bubbles?

While considerable attention has been paid on the sliding behaviors in drop transport, the surface affinity of bubbles is essential for preventing bubble detachment and transport failure, especially under dynamic or high‐speed conditions [16]. In many practical engineering systems (e.g., electrocatalysis, surface cleaning), rapid sliding of bubbles while maintaining strong affinity and low resistance is highly desirable [17, 18, 19]. However, in everyday scenarios, it is commonly observed that bubbles rarely adhere to clean, aerophobic surfaces like glassware or pool walls; but once these surfaces become contaminated and aerophilic, bubbles effortlessly attach and remain fixed. Given the uncertainty about whether bubble stickiness and bubble slipperiness can be decoupled, it remains challenging to simultaneously achieve robust attachment and ultralow resistance during bubble transport. From the nanoscopic perspective, hydrophobic interaction is considered as the pivotal driving force governing bubble attachment, thereby directly affecting the interfacial stability of bubbles on solid surfaces [20, 21]. Bubble sliding, on the other hand, fundamentally depends on friction force, which dominates the dynamics of three‐phase contact line motion [10, 22, 23]. Therefore, precisely modulating bubble‐surface interactions, i.e., hydrophobic interaction and friction force, offers a promising strategy to regulate bubble stickiness and slipperiness, thus enabling the simultaneous achievement of both robust attachment and ultralow resistance. For instance, hydrophobic nanochannels could be switched from “slippery” to “sticky” by exploiting ion‐water interactions and tuning the molecular spacing at the solid wall, offering a pathway to control fluid mobility without altering surface chemistry [24], yet remains largely unexplored in bubble‐surface interactions during bubble transport in aqueous environments.

Over the last several years, substantial efforts have been devoted to quantifying the interaction forces (e.g., van der Waals and hydrophobic interactions) between air bubbles and solid surfaces in aqueous media [20, 25]. Bubble probe atomic force microscopy (AFM), as a nanomechanical technique, has enabled the deciphering of these interactions with ultrahigh spatial resolution and force sensitivity [26, 27, 28]. Despite significant advances, however, direct and quantitative measurement of bubble‐solid friction, essential for determining bubble mobility, has remained experimentally inaccessible. Achieving this capability would allow precise detection of nanonewton‐scale variations in both hydrophobic interaction and interfacial friction, thus unlocking molecular‐level origins of bubble stickiness and slipperiness on ultra‐slippery surfaces, a long‐standing question in bubble dynamics. Meanwhile, polydimethylsiloxane (PDMS) has emerged as one of the most promising eco‐friendly coating materials for achieving low‐resistance bubble transport [29, 30, 31, 32]. The nanoscale surface characteristics (e.g., molecular order, surface heterogeneity) of PDMS coating could influence bubble transport behaviors. Nevertheless, how these nanoscale features affect the stickiness and slipperiness of bubbles, particularly for the underlying hydrophobic interaction and friction mechanisms at the nanoscale, remains poorly understood, which hinders the fundamental understanding and modulation of interaction mechanisms underlying bubble transport.

**SCHEME 1:**
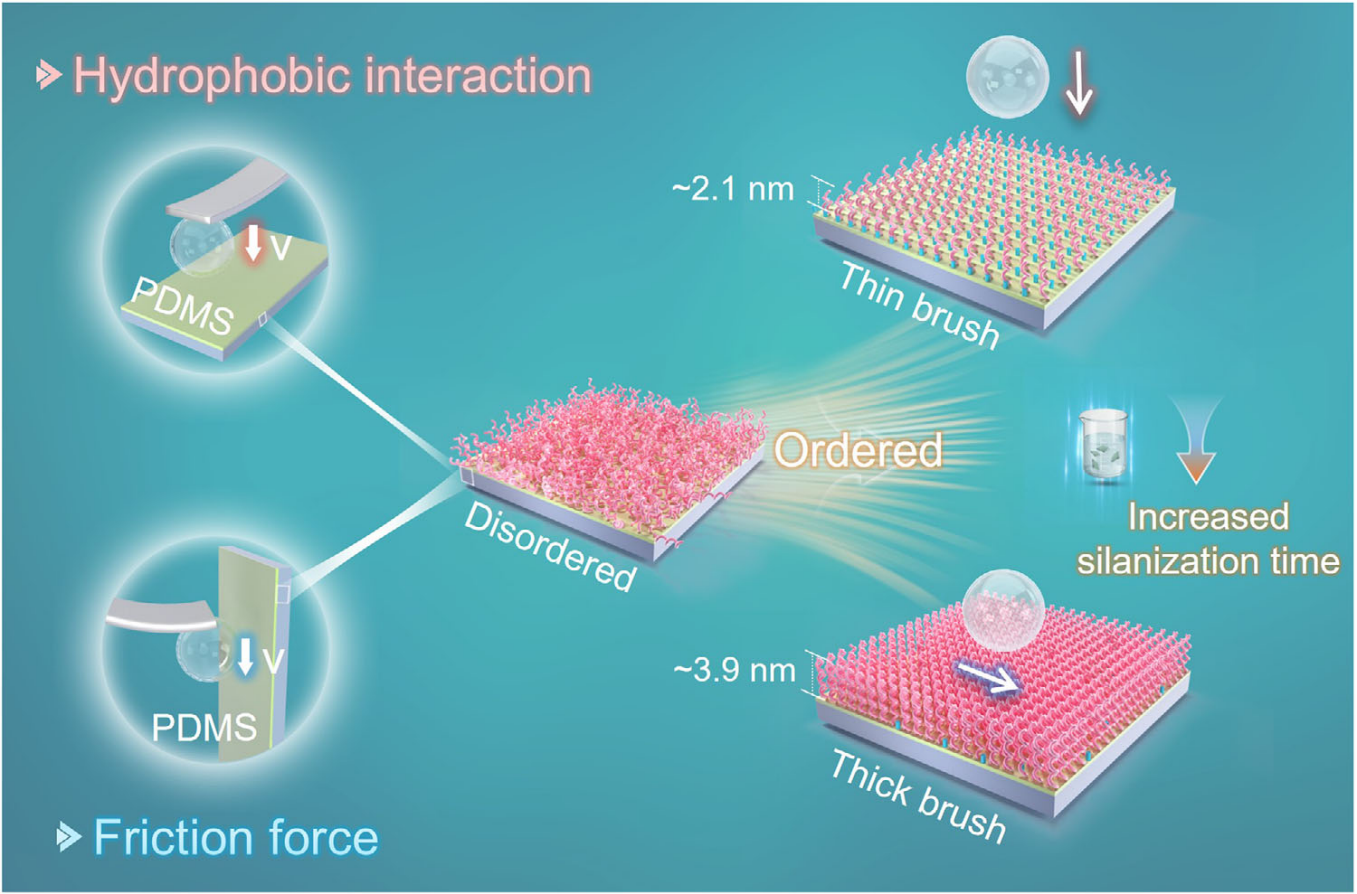
Schematic illustration of bubble probe AFM for measuring hydrophobic interaction and friction force on PDMS surfaces with different molecular architectures.

Herein, to systemically elucidate how the molecular architecture of PDMS surfaces governs the stickiness and slipperiness of bubbles at the nanoscale, bubble probe AFM was used for the first time to precisely quantify both hydrophobic interaction and friction force between air bubbles and PDMS surfaces of different molecular orders and layer thicknesses in aqueous media (Scheme 1). We used two representative types of PDMS surfaces: i) spin‐coated PDMS network featuring a disordered molecular architecture, and ii) PDMS covalently attached liquid surfaces (CAL) exhibiting ordered molecular architectures, whose layer thickness could be finely tuned from ∼2.1 to ∼3.9 nm by varying the silanization time *x* in seconds (denoted as CAL@*x*, *x* = 30, 100, 300, 400, and 1800). To the best of our knowledge, neither hydrophobic interaction nor friction force has been systematically measured for bubble‐surface interactions on these PDMS architectures. Remarkably, the strongest hydrophobic interaction and lowest friction force were attained for CAL@1800 that exhibited the most ordered and uniform molecular architecture, reconciling the stickiness and slipperiness during bubble transport. Molecular dynamics (MD) simulations further revealed that the ordered brushes induce a well‐defined interfacial depletion layer that enhances hydrophobic interaction, while the suppression of surface heterogeneity minimizes contact line pinning and energy dissipation. This study provides the first quantitative and molecular‐level understanding of how nanoscale molecular architecture simultaneously modulates bubble affinity and friction, enabling the decoupling of stickiness and slipperiness in bubble‐surface interactions. These findings establish a universal design principle for next‐generation ultralow‐resistance bubble transport interfaces, with broad implications for interfacial catalysis, microfluidic, gas‐liquid separation, and adaptive wetting systems.

## 2 Results and Discussion

### 2.1 Bubble Attachment on Disordered and Ordered PDMS Surfaces

**FIGURE 1:**
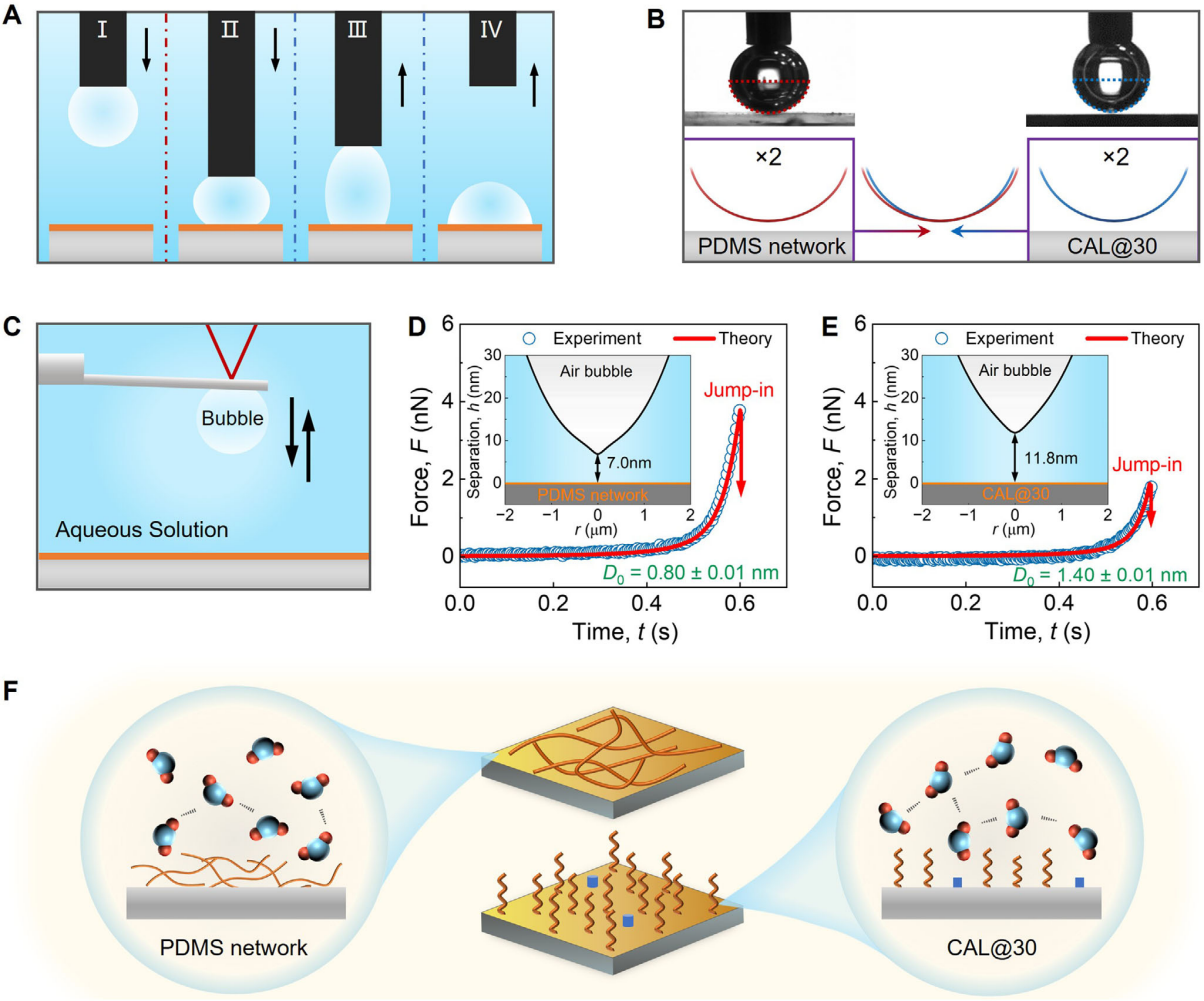
Attachment tendency and interaction of bubbles with PDMS network and CAL@30 during the approach‐retraction process. (A,B) Schematic illustration of the process of bubble (volume *V*_0_ = 1 µL) attachment to PDMS surfaces, and the extracted and magnified bubble profiles prior to bubble attachment. (C) Experimental setup of interfacial interaction measurement using bubble probe AFM (radius *R*_0_ = 55–61 µm). Bubbles of comparable size were selected to minimize the influence of bubble size on the measured forces. (D,E) Measured interaction forces (blue open symbols) and theoretically fitting results (red solid curves). The inset shows the calculated bubble profiles prior to attachment. (F) Schematic illustration of the chain configurations (center) of PDMS surfaces and their contact with water molecules: PDMS network (left) and CAL@30 (right). The blue columns on the surface represent surface hydroxyl groups ungrafted with PDMS chains.

Bubble stickiness on a solid surface is closely associated with the surface affinity toward bubble, which can be qualitatively inferred from the bubble profile during attachment. Here, we first compared the affinity of bubbles to two representative PDMS surfaces with different molecular orders (disordered PDMS network and ordered CAL@30) in aqueous media. As illustrated in Figure 1A and Movies S1 and S2, the bubble generated at the capillary orifice was driven to impact PDMS surfaces, forming a capillary bridge at the three‐phase interface (state I‐II), which subsequently ruptured during separation and caused the bubbles to detach from the capillary (state III‐IV). Prior to bubble‐surface contact, the bubble profile was stretched more sharply for CAL@30 compared to PDMS network (Figure 1B), which may indicate the stronger bubble affinity for CAL@30. It was noted that this profile distortion may be influenced by the bubble pinning at the needle.

To verify the above disparity in bubble affinity between the two surfaces, the interaction forces between air bubbles and PDMS surfaces in aqueous media were quantitatively measured using bubble probe AFM (Figure 1C), with the force profiles shown in Figure 1D,E. To isolate the contribution of hydrophobic interaction, a high‐salinity electrolyte was used to screen the electrical double layer interaction, allowing the hydrophobic contribution to dominate the overall surface forces. As the bubble approached the surfaces, a repulsive force was registered ascribed to van der Waals and hydrodynamic interactions, with magnitudes of ∼3.8 nN for PDMS network and ∼1.8 nN for CAL@30, and subsequently the immediate bubble attachment was detected as indicated by the “jump‐in” behavior. The measured force curves were quantitatively analyzed using the Stokes‐Reynolds‐Young‐Laplace (SRYL) model incorporated with a hydrophobic disjoining pressure term $\Pi_{\mathrm{HB}}=-\frac{\gamma (1-\cos\theta_{W})}{D_{0}}e^{-h/D_{0}}$, where *γ* is the water surface tension, *h* is the bubble‐surface separation, and *D*_0_ denotes the decay length of hydrophobic interaction. The fitted *D*_0_ values were ∼1.40 nm for CAL@30 and ∼0.80 nm for PDMS network, revealing a stronger hydrophobic attraction between the bubble and the ordered CAL@30 surface.

The calculated disjoining pressure‐separation profiles (Figure S1) further corroborate these findings. For both surfaces, the overall disjoining pressure *Π*_Overall_ turned attractive and progressively increased with the reduced separation, nearly overlapping with the hydrophobic disjoining pressure *Π*_HB_, reflecting the dominant role of hydrophobic interaction in bubble attachment. At the central separations of ∼7.0 and ∼11.8 nm, *Π*_Overall_ exceeded the Laplace pressure and the “jump‐in” occurred for PDMS network and CAL@30, respectively. Based on the SRYL theoretical model, the bubble profiles at the critical separation were reconstructed from the AFM force‐distance curves (insets in Figure 1D,E), thereby enabling the visualization of bubble deformation immediately prior to attachment. The reconstructed bubble profiles displayed pronounced “pimple” shapes, as the bubble bottom was pulled toward the surfaces prior to bubble attachment. On CAL@30, a larger critical separation and a more conspicuous bubble pimple were observed, suggesting a stronger propensity for bubble attachment, despite its lower surface hydrophobicity than PDMS network. Specifically, CAL@30 exhibited a static water contact angle (*θ*_W_) ≈96° and a static underwater air contact angle (*θ*_A_) ≈86°, compared with *θ*_W_ ≈102° and *θ*_A_ ≈78° for PDMS network (Figures S2 and S3). This reversal reveals that surface wettability alone cannot fully capture the strength of bubble attachment, and additional nanoscale interactions play a decisive role. Importantly, this work distinguishes between surface hydrophobicity, reflected by static water contact angle, and hydrophobic interaction, reflected by nanoscale interaction force—two parameters that are often conflated but not equivalent.

Although both PDMS‐based surfaces share identical chemical composition, the hydrophobic interaction with bubbles differed substantially between the disordered PDMS network and the ordered CAL@30. This raises an intriguing question: What governs the stronger hydrophobic interaction on CAL@30 despite its apparent lower macroscopic surface hydrophobicity? Our previous findings reveal that hydrophobic interaction can be modulated by tuning molecular order, where a transition from disordered to ordered arrangement leads to a strengthened decay length *D*_0_ from ∼0.80 to ∼1.40 nm due to reduced molecular heterogeneity [21, 33]. Inspired by this principle, the distinct hydrophobic interactions for PDMS network and CAL@30 could be attributed to their different interfacial water layers induced by molecular configurations (Figure 1F), independent of the macroscopic contact angle. The formation of heterogeneous clusters and the random occurrence of entanglement constraints make the molecular arrangement of PDMS network dominated by frozen‐in disorder [34, 35]. Due to molecular self‐closure, the Si─O─Si backbone adopts a slightly twisted alternating trans‐cis conformation [36]. In contrast, the covalently grafted PDMS chains in CAL@30 experience steric hindrance that drives them to extend away from the substrate, thereby forming an ordered “standing” configuration [37, 38, 39]. The ordered molecular arrangement minimizes surface heterogeneity and promotes the formation of a more ordered interfacial water structure, which leads to a larger *D*_0_ value and stronger bubble affinity, even with lower surface hydrophobicity.

### 2.2 Bubble Sliding on Disordered and Ordered PDMS Surfaces

**FIGURE 2:**
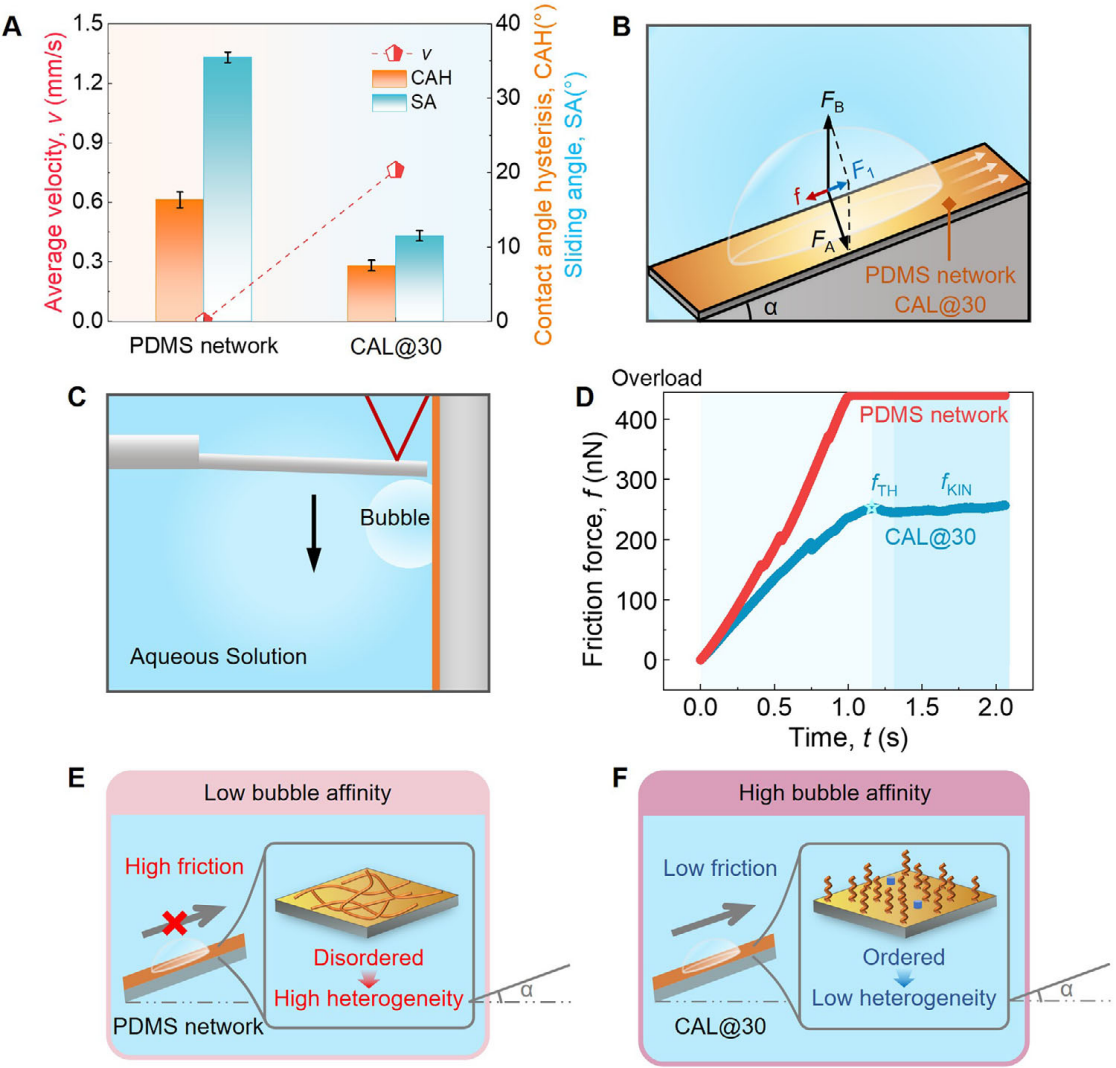
Transport behavior and friction of bubbles sliding on PDMS network and CAL@30. (A) Comparison of bubble transport behaviors, where the selected bubble sizes for measuring the sliding velocity and CAH/SA were 20 µL and 5 µL, respectively. (B) Schematic illustration of the force analysis on underwater bubbles sliding along the tilted PDMS surfaces, where *F*_B_ is the buoyancy force, *F*_A_ is the adhesion force, *F*_1_ is the driving force, and *f* is the total resistant force. The inclination angle *α* is fixed at 20°. (C) Experimental setup of friction force measurement using bubble probe AFM (radius *R*_0_ = 20–25 µm, contact width *w* = 36–45 µm). Bubbles of comparable size were selected to minimize the influence of bubble size on the measured forces. (D) Characteristic friction force curves versus time. The three green backgrounds from light to dark indicate three phases of bubble friction, namely the stationary phase, transitional phase, and kinetic phase. (E,–F) Schematic illustration of the effect of molecular order and heterogeneity on friction force during the sliding of bubbles. The blue columns on the surface represent surface hydroxyl groups ungrafted with PDMS chains.

To elucidate how molecular order influences another key aspect of bubble transport, namely sliding, the motion of bubbles on PDMS network and CAL@30 was examined (Figure 2A). Due to the larger contact angle hysteresis (CAH, defined as the difference between advancing and receding contact angles) and sliding angle (SA), bubbles on PDMS network remained pinned without any observable motion, whereas they slid readily on CAL@30 with an average velocity of approximately 0.76 mm/s. This pronounced contrast in mobility can be rationalized by considering the balance of forces acting on the bubbles (Figure 2B, and detailed analysis in Note S1). The identical driving force *F*_1_ was sufficient to overcome the frictional resistance on CAL@30 but insufficient on PDMS network, leading to bubble sliding only on the former. This difference can be explained according to the Furmidge‐Kawasaki equation (Equation S3) [40, 41], which relates sliding resistance to the difference between the advancing and receding contact angles. According to this relation, a larger contact angle hysteresis corresponds to stronger contact line pinning and therefore higher frictional resistance. Considering the comparable size and velocity of sliding bubbles on both surfaces, the hydrodynamic drag f_D_ is nearly identical. Therefore, the frictional resistance of bubble on PDMS network can be infered higher than that on CAL@30. Remarkably, CAL@30 simultaneously exhibited strong bubble affinity and low friction, two properties typically considered mutually exclusive. All surfaces were confirmed to be molecularly smooth, with root‐mean‐square (rms) roughness below 0.5 nm (Figure S4), suggesting that surface heterogeneity was limited exclusively to the molecular‐level organization rather than topographic defects, which may exert opposing effects on fluid friction [42].

To quantify the influence of molecular order on frictional resistance directly, bubble‐surface friction forces were measured using AFM (Figure 2C). And the force results are consistent with the predictions of the Furmidge–Kawasaki equation. On PDMS network, the bubble remained immobilized until the cantilever exceeded the thrust limit (Figure 2D), indicating that the static friction force was considerably stronger than the applied load. Under identical measurement conditions, the bubble on CAL@30 began sliding once the threshold force (*f*_TH_, ∼252.3 nN) was reached and subsequently maintained steady motion with the kinetic friction force *f*_KIN_, ∼249.9 nN after a brief transitional regime (Figure 2D). The high friction observed for PDMS network could be attributed to the presence of randomly distributed entanglement constraints and cluster structures within its disordered molecular architecture [43]. Furthermore, as a soft, cross‐linked elastomer, PDMS network is susceptible to viscoelastic deformation under the stress exerted by the moving three‐phase contact line [44]. This additional viscoelastic dissipation likely contributes to the enhanced contact angle hysteresis and frictional resistance observed on PDMS network [45]. In contrast, CAL@30 comprises covalently attached Si─O─Si backbones whose glass transition temperature lies below room temperature, rendering the brush layer dynamically mobile and liquid‐like [31]. This liquid‐like property effectively suppresses chain entanglement and promotes a more ordered and uniform interfacial configuration, which minimizes the frictional dissipation [38]. Consequently, the disordered PDMS network, despite its weak bubble affinity, exhibited strong frictional resistance; while the ordered CAL@30 displayed high bubble affinity coupled with remarkably low friction (Figure 2E,F). These results demonstrate that molecular ordering governs bubble friction by tuning interfacial chain mobility, substrate deformation and surface homogeneity, thereby enabling the coexistence of strong attachment and facile sliding.

### 2.3 Bubble Attachment and Sliding on Increasingly Ordered PDMS Surfaces

In view of the strong hydrophobic interaction and low friction observed between bubble and CAL@30, it is essential to further explore how polymer layer thickness of CAL surfaces influences bubble attachment and sliding dynamics. Therefore, the silanization duration was systematically varied until reaction completion, providing CAL surfaces with thickness ranging from ∼2.1 to ∼3.9 nm (Figures S5–S7). All CAL surfaces exhibited the similar attachment behavior: bubbles were first attracted to the surface and then spread upon contact. However, the time interval between initial attachment and subsequent spreading decreased markedly with the elevated silanization time, from ∼0.42 s for CAL@30 to ∼0.06 s for CAL@300 and ∼0.05 s for CAL@1800 (Movies S2 and S3; Figures S8–S10). This trend could indicate a progressively stronger bubble affinity for thicker SOCAL layers. Consistently, the steady sliding velocity increased from 0.76 mm/s (CAL@30) to nearly 4.22 mm/s (CAL@1800), accompanied by a reduction in CAH and SA to 6.8° and 4.1°, respectively (Figure S11). Clearly, longer silanization durations yield CAL surfaces with enhanced bubble affinity and reduced sliding resistance. Notably, despite the extremely low CAH exhibited by CAL@1800, droplet sliding velocity remains moderate, likely due to the hindered contact line dynamics arising from interactions between water molecules and the terminal silanol groups of PDMS chains [46, 47].

**FIGURE 3:**
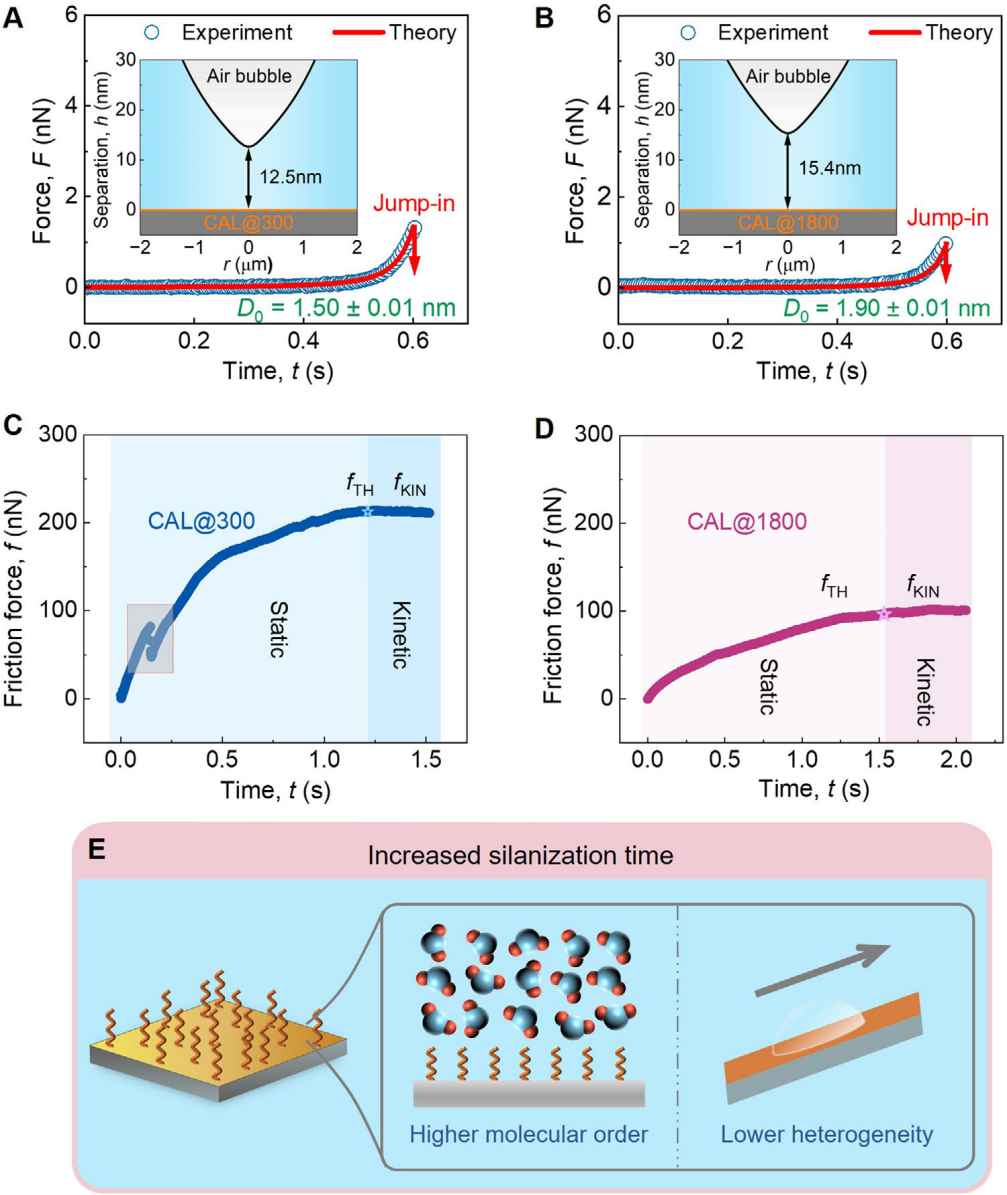
Nanoscale force results during bubble attachment and sliding on PDMS CAL@300 and CAL@1800. Bubbles of comparable size were selected to minimize the influence of bubble size on the measured forces. (A,B) Interaction forces between bubbles (*R*_0_ = 52–58 µm) and CAL surfaces during the approach‐retraction process. (C,D) Characteristic friction force curves versus time for bubbles (*R*_0_ = 20–25 µm, *w* = 36–45 µm) sliding on CAL surfaces. The light and dark backgrounds indicate the transitional and kinetic phase, respectively. (E) Schematic illustration of the chain configuration of CAL surface with increased silanization time and its effect on contact with water molecules and sliding of bubbles.

To elucidate the molecular origins of this thickness‐dependent behavior, bubble‐surface interaction forces were quantified using AFM for representative CAL surfaces (CAL@300 and CAL@1800). Figure 3A,B shows the measured force curves and theoretical fittings. Compared to CAL@30, the fitted *D*_0_ value of hydrophobic interaction increased from ∼1.40 to ∼1.50 nm for CAL@300 and ∼1.90 nm for CAL@1800, with the corresponding critical separation before attachment increasing from ∼11.8 to ∼12.5 nm and ∼15.4 nm (Figure S12), respectively. These results demonstrate that thicker PDMS brush layers enhance the hydrophobic interaction and surface affinity between bubbles and CAL surfaces. This observation is consistent with previous reports by Zhou et al. [38], who showed that polymer chains become increasingly ordered and uniformly cover the substrate with the elevated layer thickness, as evidenced by the symmetric/antisymmetric stretch ratio in sum frequency generation (SFG) spectroscopy. It is noteworthy that surface hydrophobicity also increased moderately with silanization time, rising from ∼96° (CAL@30) to ∼100° (CAL@300) and ∼102° (CAL@1800) (Figure S2). Thus, the incremental thickness of PDMS brush layer enhanced both surface hydrophobicity and hydrophobic interaction, in contrast to the behavior observed between PDMS network and CAL@30.

During bubble sliding, the kinetic friction force decreased significantly from ∼249.9 nN for CAL@30 to ∼212.7 nN for CAL@300 and further to ∼99.9 nN for CAL@1800 (Figure 3C,D). To eliminate the influence of contact geometry, we normalized the measured friction forces by the contact width extracted from bubble profiles. After normalization, the observed trends across different surfaces remained unchanged (Figure S13). This trend aligns with the changes in the advancing and receding contact angles of bubbles on surfaces, as predicted by the Furmidge–Kawasaki equation. Notably, bubbles on CAL@300 and CAL@1800 transitioned directly into the kinetic state without a discernible transitional phase, which could be attributed to the enhanced flexibility of thicker PDMS brush layers, which enables rapid conformational adaptation of polymer chains to the moving contact line [48]. Interestingly, the stick‐slip motions were evident on both CAL@30 and CAL@300 (Figure S14) but completely absent on CAL@1800, providing direct evidence of surface defects on the two former surfaces [22]. These defects likely arise from the structural imperfections and discontinuous chain coverage at low layer thickness [38]. Moreover, PDMS chains are partially disordered within thin layers, leading to molecular‐scale vacancies that allow air molecule penetration into the interface [10, 49, 50]. The trapped air patches act as localized pinning sites for the three‐phase contact line, thereby amplifying frictional resistance and causing intermittent stick‐slip behavior. With increasing silanization time, the order and density of PDMS brush layer increase, effectively eliminating these interfacial vacancies (Figure 3E). The resulting highly ordered and defect‐free CAL@1800 surface provides a uniform and whole liquid‐like interface that allows the contact line to move smoothly with minimal energy dissipation. Consequently, both surface heterogeneity and interfacial friction are drastically reduced, enabling the simultaneous realization of strong bubble affinity and ultralow sliding resistance.

Moreover, the disparity between *θ*_W_ and *θ*_A_ decreased with shortened silanization durations. Specifically, CAL@1800 exhibited a pronounced *θ*_W –_ *θ*_A_ difference of 25° (102° vs 77°, Figures S2 and S3), which was substantially diminished to 10° for CAL@30 (96° versus 86°, Figures S2 and S3). This trend reflected an interfacial response to the surrounding medium, induced by the conformational rearrangement of flexible PDMS chains terminated with hydrophilic silanol groups [46, 47, 51]. When contact with water, the terminal silanol groups preferentially partition toward the interface, whereas in contact with air they are partially buried within the brush layer. Relative to surfaces lacking chain rearrangement, this environment‐dependent reorganization renders the surface more hydrophilic in aqueous contact, while promoting aerophilicity upon bubble attachment. With the prolonged silanization duration, the tethered chains attain higher molecular weight and the surface density of silanol groups concomitantly decreases, progressively attenuating this effect. This silanol‐induced, environment‐responsive interfacial heterogeneity could also offer a molecular‐level explanation for the observed differences in friction forces. In shorter brushes (e.g., CAL@30), a higher effective interfacial concentration of silanol groups might create more pinning sites, thereby hindering contact line motion and contributing to frictional dissipation [46]. In contrast, the diluted and dynamically shielded silanols in the thicker and more ordered brushes (e.g., CAL@1800) could minimize such pinning events, thereby enabling low‐friction bubble sliding [47].

**FIGURE 4:**
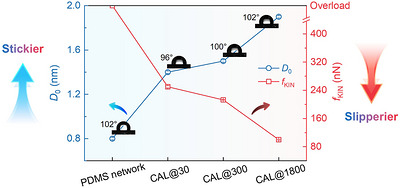
Summary of the quantitative results of hydrophobic interaction and friction force of bubble‐PDMS system. The blue dots represent the decay length *D*_0_ of hydrophobic interaction, and the red squares denote the kinetic friction force *f*_KIN_ during bubble movement. The insets show the water contact angle images of corresponding surfaces.

Figure 4 summarizes the quantitative relationship between hydrophobic interaction and friction force during bubble attachment and sliding across all examined surfaces. Conventionally, surfaces with larger *θ*_W_ are expected to exhibit stronger hydrophobic interactions with bubbles in aqueous media, thereby promoting bubble affinity [52]. In contrast to this empirical expectation, the brush‐like CAL@30, despite its lower surface hydrophobicity (*θ*_W_ ∼96°), displayed a markedly stronger hydrophobic interaction (*D*_0_ ∼1.40 nm) than PDMS network (*θ*_W_ ∼102° and *D*_0_ ∼0.80 nm). This enhancement originates from the more ordered molecular configuration of CAL@30. In parallel, CAL@30 also exhibited a significantly reduced frictional response, indicating that ordered molecular architecture favors both strong affinity and low resistance. When the silanization time was extended, both the hydrophobicity and hydrophobic interaction of CAL surfaces increased, while the friction force decreased progressively, a trend attributed to the improved molecular order and reduced heterogeneity. Among all tested samples, CAL@1800 possessed the most ordered and homogeneous polymer structure, yielding the largest decay length (*D*_0_ ∼1.90 nm) and the lowest kinetic friction force (*f*_KIN_ ∼99.9 nN). This finding represents a striking contrast to the disordered PDMS network, which exhibited comparable hydrophobicity yet much weaker hydrophobic interaction and substantially higher friction. Overall, these results reveal a clear inverse correlation between molecular order/homogeneity and friction, and a direct correlation between molecular order/homogeneity and hydrophobic interaction strength. The systematic modulation of nanoscale molecular architecture thus provides a unified framework to reconcile the stickiness and slipperiness in bubble‐surface interactions.

### 2.4 Molecular Mechanisms Underlying This “Sticky‐Slippery” Synergy

**FIGURE 5:**
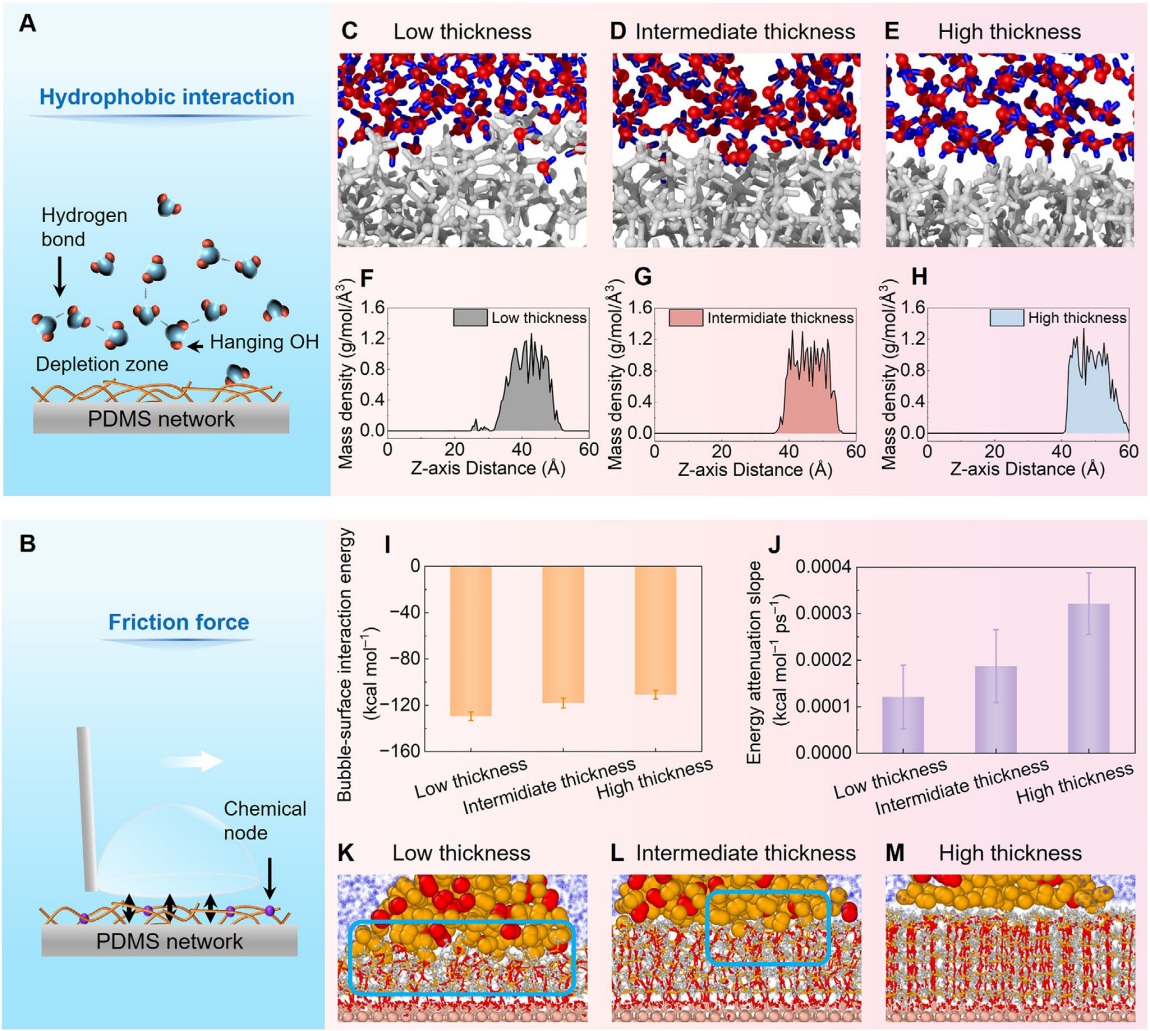
Schematic illustration and MD simulations depicting the mechanisms underlying bubble attachment (top) and sliding (bottom) at the PDMS‐water interface. (A,B) The molecular chains on PDMS network are entangled to form a crosslinked network, which (A) disorders the arrangement of interfacial water molecules and (B) restricts the sliding of bubbles. (C–E) Locally enlarged view from MD simulations showing details of interfacial water molecules and their arrangement as a function of brush layer thickness. (F–H) Mass density profiles of water along the surface‐normal (z) direction for low (F), intermediate (G), and high (H) brush thickness. (I,J) Bubble‐surface interaction energy (I) during steady sliding and the fitted energy attenuation slopes (J) for low, intermediate, and high brush thickness. (K–M) Locally enlarged view of bubble at initial sliding on PDMS brushes of low (K), intermediate (L), and high (M) thickness, illustrating the molecular brush arrangement and pinning sites (highlighted by the blue rectangles).

Having established that molecular order and homogeneity decisively regulate both the hydrophobic interaction and friction force, a key question arises: what are the underlying molecular‐scale mechanisms? The observed disparities among PDMS surfaces can be rationalized in terms of interfacial water structuring and energy dissipation dynamics, rather than macroscopic wetting alone. At the solid–liquid interface, water molecules near hydrophobic surfaces must disrupt their hydrogen‐bond network, producing free‐hanging hydroxyl groups oriented away from the bulk phase [53]. These dangling OH groups induce nearby water molecules to reorganize into an ordered and low‐density configuration, forming a water‐vapor‐like depletion zone that promotes dipole pairing adjacent to hydrophobic interface [54]. The thickness of this depletion zone provides a molecular‐scale indicator of hydrophobic interaction strength [55]. For the disordered PDMS network, interfacial water molecules exhibit heterogeneous and weakly correlated orientations (Figure 5A), arising from the loose physical constraints of entangled chains. This fragmented hydration structure limits the spatial extent of the depletion zone, resulting in a weaker exponential attraction and hence a shorter‐ranged hydrophobic interaction [38, 56]. In addition, the chain configuration strongly influences the mode of energy dissipation during bubble sliding (Figure 5B) [57]. Within the PDMS network, molecular entanglements and chemical nodes act as frictional constraints that hinder interfacial chain motion and dissipate substantial energy, leading to the large observed friction force [58, 59].

The molecular origins of the bubble sticky‐slippery behavior on CAL surfaces of varying thickness were further visualized by MD simulations performed using LAMMPS (Figure 5; see more details in Figures S15–S21). Compared to PDMS network, the chains of CAL@30 transition from disordered entanglement to an ordered, brush‐like configuration oriented perpendicularly to the substrate (Figure 5C), which accounts for the enhanced hydrophobic interaction. However, the low‐thickness brush layer still exhibits residual interfacial irregularity due to ungrafted surface hydroxyl groups, allowing partial water accessibility and resulting in a diffuse interface with a poorly defined depletion layer. With extending silanization time, the brush becomes thicker and more densely grafted, forming a more ordered layer that effectively suppresses the accessibility of water molecules (Figure S22A; Figure 5D,E).

Mass density profiles along the surface‐normal (z) direction reveal that water accessibility into the brush layer is progressively suppressed (Figure 5F–H), leading to the emergence of a distinct depletion region at high brush thickness. This suppression of water accessibility likely arises from the decreased water‐CAL interaction energy, from −330.56 ± 20.71 kcal mol^−1^ for the low thickness system to −176.88 ± 9.23 kcal mol^−1^ at high thickness (Figure S22B). Moreover, increasing brush thickness gradually reduces the diffusion coefficient of interfacial water, from 5.22 × 10^−8^ to 4.27 × 10^−8^ cm^2^ s^−1^ (Figure S22C), indicating enhanced confinement of water molecules near the thicker brush layer. Further analysis of orientational distribution of interfacial water molecules, quantified by cosα (the angle between the hydroxyl bond vector and the surface normal), reveals a systematic transition from negative values (−0.8 to −1.0) at low thickness to positive values (0.1 to 0.5) at high thickness (Figure S22D), indicating that hydroxyl groups progressively orient away from the surface. The resulting hanging hydroxyl groups and expanded depletion zone lead to the increase in hydrophobic interaction decay length. This effect is most pronounced for CAL@1800 (Figure 5E), demonstrating that the observed increase in *D*_0_ originated from changes in interfacial water structuring rather than macroscopic wettability.

The slip behavior at interface is determined not only by the intrinsic wettability, but also by how shear is distributed across the confined region and how it couples with the local molecular arrangement [60]. MD simulations revealed that the bubble sliding distance over the same duration, viz., bubble sliding velocity, increases markedly with PDMS brush thickness, as shown in Figures S23 and S24), in quantitative agreement with experimental observations. To further elucidate the underlying mechanism, the energy dissipation were quantified by analyzing the temporal evolution of bubble‐surface interaction energy within the steady sliding regime (Figure 5I,J). The time‐averaged interaction energies decrease from –129.30 ± 3.64 kcal mol^−1^ for low brush thickness, −117.99 ± 4.23 kcal mol^−1^ for intermediate brush thickness, and −110.80 ± 3.74 kcal mol^−1^ for high brush thickness (Figure 5I), suggesting diminished interfacial coupling and lower sliding resistance in thicker brushes. Analysis of the temporal attenuation of interaction energy shows that the decay slopes follow the order (Figure 5J): high thickness (∼3.21 × 10^−4^ kcal mol^−1^ fs^−1^) > intermediate thickness (∼1.87 × 10^−4^ kcal mol^−1^ fs^−1^) > low thickness (∼1.21 × 10^−4^ kcal mol^−1^ fs^−1^), indicating faster relaxation of interaction energy on thicker brush layers. This trend arises from the dense and ordered chains in thicker brushes, which reduce bubble‐surface interaction energies, hinder gas penetration into the layer, and accelerate energy relaxation, thereby minimizing frictional dissipation. Conversely, in thin brushes, gas penetration promotes more intimate contact with PDMS chains, slowing energy relaxation and increasing resistance to bubble motion.

To probe the “stickiness” of thick brush layers, MD simulations were performed to examine bubble detachment from surfaces with different brush thickness under an identical upward driving force (Figure S25). Representative structural snapshots (Figure S25A, inset) show that the bubble remains attached to the thick brush layer throughout the simulation, whereas on the brush of low thickness it progressively detaches and rises into the liquid phase. Consistently, the bubble velocity along the *z* direction (Figure S25B) remains nearly zero on the thick layer (0.00002 ± 0.00006 Å fs^−1^), while the bubble on thin brush layer rises with a velocity of 0.00139 ± 0.00016 Å fs^−1^. These results provide direct microscopic evidence that thick and ordered PDMS brush layers suppress bubble detachment, giving rise to the “sticky” characteristic of the interface.

These detachments and sliding simulations described above highlight the dual role of brush architecture in regulating bubble adhesion and friction. The above observations are consistent with the argument that disordered architecture in thin brushes, grafted at low packing densities, contribute to energy dissipation by creating interfacial vacancies that trap air molecules and act as pinning sites for contact line movement (Figure 5K,L). These disordered layers undergo local conformational rearrangements during sliding, lacking the coherent domain‐level motion observed in thicker brushes and thus producing higher friction and energy loss (see more details in Movies S4 and S5). These findings are consistent with recent molecular dynamics simulations reported by Rasera et al. [61], who demonstrated that chemical heterogeneity and nanoscale defects arising from incomplete surface coverage at low grafting densities significantly enhance contact line pinning and hysteresis in tethered polymer layers. In particular, their work highlights the key role of exposed substrate regions and interfacial structural disorder in promoting dissipation during interfacial motion. In our system, the presence of hydroxyl groups ungrafted with PDMS chains in thin brush layers mainly plays a similar role. In contrast, the ordered and thick PDMS brushes (CAL@1800) exhibit a liquid‐like cohesive layer that imposes minimal shear resistance. As shown in Figure 5M, the dense and ordered molecular arrangement provides a structural basis for ultra‐low friction [32, 62, 63]. Upon shearing, the relatively large molecular domains tilt and reorient, maintaining the packing density and chain order with only minor volumetric fluctuation (see more details in Movie S6). This coherent molecular motion reduces both structural heterogeneity and localized deformation, thereby minimizing energy dissipation [64]. These results also align with theoretical and simulation predictions that increased interfacial homogeneity and reduced local deformation favor stable, low‐dissipation sliding [61]. Consequently, increasing thickness and grafting density promotes molecular ordering, which reduces nanoscale heterogeneity and suppresses pinning sites at the contact line, ultimately lowering frictional dissipation. Energy dissipation is therefore significantly higher in thinner PDMS layers, particularly near hydroxyl groups ungrafted with PDMS brushes; whereas thicker and more ordered brush layers provide a uniform and defect‐free interface that supports smooth, low‐friction bubble motion.

Overall, these findings demonstrate that molecular order primarily governs hydrophobic interactions and bubble affinity, whereas surface heterogeneity controls friction through contact line pinning. The detachment simulations further confirm that thick and ordered PDMS brush layers generate strong bubble‐surface adhesion that suppresses bubble release, corresponding to the “sticky” characteristic of the surface. At the same time, the homogeneous and defect‐free brush structure minimizes contact line pinning and interfacial energy dissipation during sliding. The decoupling of these two effects, both regulated by the structural evolution of the PDMS brush layers, enables the coexistence of strong bubble affinity and ultralow friction, which underlies the observed “sticky‐slippery” behavior in bubble‐surface interactions.

Due to strong intermolecular interactions, gas bubbles typically adhere to solid surfaces and stay in place. How, then, does CAL@1800 simultaneously exhibit the highest bubble affinity yet the lowest friction? This coexistence indicates that bubble friction cannot be solely governed by the strength of bubble‐surface affinity, but rather arises from multiple effects, including hydrodynamic drag and contact line friction [22, 23]. Given that hydrodynamic contributions remain invariant for bubbles of comparable size and velocity, the observed friction reduction should be ascribed to the differences in contact line friction. In the low‐velocity regime (typically below 0.01 m/s, in the present study, 5 µm/s fall within this regime), the pinning‐depinning events arising from nanoscale surface heterogeneity may dominate the dynamics of three‐phase contact line [65, 66]. Increasing silanization time leads to a higher grafting density and improved chain ordering of the PDMS brush layers. This structural evolution reduces molecular‐scale heterogeneity and minimizes exposed hydrophilic defects, which otherwise serve as strong pinning sites. The minimum defects capable of inducing pinning are of molecular dimensions, and their influence diminishes with increasing surface uniformity [67]. As evidenced by our MD simulations, the clusters of gas molecules at the contact line initially advanced coherently but were intermittently trapped at large defect sites (e.g., PDMS network and CAL@30; see Movie S4 for details), acting primarily as metastable pinning centers and forming a locally pinned three‐phase contact line. From a thermodynamic perspective, each depinning event required additional displacement to overcome the corresponding energy barrier via thermal activation, the magnitude of which depended on the strength of contact line attachment to localized hydrophilic sites [68]. Importantly, the spatial discontinuity of these trapped gas clusters prevented the formation of a coherent lubricating film. Instead, the advancing contact line underwent repeated transitions between pinned states and localized slip, thereby increasing frictional dissipation during sliding. In contrast, thick PDMS brush layers with enhanced molecular order and reduced heterogeneity (e.g., CAL@1800) contained far fewer hydrophilic defects, resulting in both lower pinning frequency and weaker pinning strength (Movies S5 and S6). Correspondingly, the energy barriers that impeded contact line motion were markedly reduced, leading to minimal frictional dissipation and smooth bubble sliding. Therefore, the molecular‐scale suppression of contact line pinning arising from enhanced structural homogeneity provides the physical origin for the coexistence of strong affinity and ultralow friction on ordered PDMS brush surfaces. Although extrapolation to higher speeds may introduce additional hydrodynamic dissipation of varying extents, the tendency of order arrangement to suppress pinning‐related friction is likely to remain operative, as it originates from reduced interfacial heterogeneity and fewer localized energy barriers.

## 3 Conclusion

In this work, using PDMS substrates as a representative soft interface model, we quantitatively elucidated, for the first time, how molecular architecture governs bubble‐surface interactions. Our study reveals that molecular order and surface homogeneity play decisive roles in modulating both bubble affinity and mobility. Through bubble probe AFM measurements coupled with molecular dynamics simulations, we demonstrated that the transition from a disordered PDMS network to ordered, covalently grafted PDMS brush layers fundamentally reconfigures the interaction landscape. The disordered PDMS network, dominated by chain entanglement and nanoscale heterogeneity, exhibited short‐ranged hydrophobic interaction and high friction force, leading to weak bubble attachment and limited mobility. In contrast, the ordered PDMS brushes, particularly the thick CAL@1800, displayed an extended hydrophobic interaction range and ultralow friction, enabling the unprecedented coexistence of strong affinity and facile sliding. At the molecular level, this “sticky‐slippery” synergy originates from the ordering‐induced suppression of nanoscale heterogeneity and contact line pinning. Molecular ordering reorganizes interfacial water molecules into a well‐defined interfacial depletion layer that enhances hydrophobic interaction, while the homogeneous and coherent reorientation of tethered polymer chains during bubble motion minimizes energy dissipation at the contact line. These molecular‐level effects decouple bubble affinity and mobility, achieving both stickiness and slipperiness demonstrated on PDMS‐based surfaces and, more generally, on other soft interfaces. This study provides the first molecular‐level evidence that bubble stickiness and slipperiness can coexist in harmony. Beyond advancing the fundamental understanding of gas‐liquid‐solid interfacial physics, these findings establish a general paradigm for rational design of adaptive, low‐dissipation soft interfaces, which offers new strategies for controlling bubble dynamics in a wide range of applications, including heterogeneous catalysis, electrochemical gas evolution, microfluidic manipulation, and separation processes.

## 4 Methods

### 4.1 Materials

Dichlorodimethylsilane (DCDMS, 99.5%), toluene (ACS reagent grade, ≥99.5%), and Sodium chloride (NaCl, ACS reagent grade, ≥99.5%) were purchased from Sigma–Aldrich. Sylgard‐184 was obtained as a two‐part kit composed of pre‐polymer and cross‐linker from Dow Corning Corporation. Toluene was obtained from Aladdin Chemical Co. Ltd, Shanghai, China. The NaCl aqueous solution was prepared using Milli‐Q water (Millipore deionized, ≥18.2 MΩ·cm resistivity) and used immediately for subsequent experiments after the ultrasonic degassing. All chemicals were used as received without further purification.

### 4.2 Preparation of PDMS Network and PDMS CAL Surfaces

Both the PDMS network and PDMS CAL surfaces were grown on silicon wafers that were previously treated with a UV‐Ozone cleaning system (100 W, SC‐UV‐I). PDMS network was prepared by spin coating PDMS solution (∼50 µL) onto silicon substrates at 2000 rpm, followed by drying under vacuum overnight to remove the remaining solvent. PDMS solution (0.5 wt.%) was prepared by dissolving PDMS elastomer in toluene for PDMS surface preparation.

PDMS CAL surfaces were prepared using the “grafting‐from” method (Figure S26) [34]. To synthesize PDMS brushes, the wafers were immersed in a reactive solution consisting of DCDMS (0.05 mL) and toluene (9.95 mL, saturated with water), and then tilted to remove excessive solution, thoroughly cleaned with Milli‐Q water, and blow‐dried with nitrogen. PDMS CAL surfaces with the treatment time of 30, 100, 300, 400, and 1800 s were defined as CAL@30, CAL@100, CAL@300, CAL@400, and CAL@1800, respectively.

### 4.3 Thickness Characterization of PDMS Brush Layers

The thickness of grafted PDMS brush layers was determined by a combination of AFM and ellipsometry. AFM force‐distance curves were acquired in the force spectroscopy mode of MFP‐3D AFM (Asylum Research, Santa Barbara) equipped with a silicon probe (AC240TS‐R3, Oxford), which allowed the estimation of PDMS brush layer thickness. Ellipsometric measurements were performed using an ellipsometer (UVISEL Plus, HORIBA France SAS, France). For each silanization duration, three independently prepared samples were characterized. For each sample, at least five distinct spots were measured and the average value was reported.

### 4.4 Surface Morphology Characterization

The morphologies of PDMS network and CAL surfaces were characterized in the tapping mode of MFP‐3D AFM (Asylum Research, Santa Barbara) using an AFM silicon probe (AC240TS‐R3, Oxford).

### 4.5 Contact Angle Measurements

Surface hydrophobicity and bubble affinity of PDMS network and CAL surfaces were assessed by the static water contact angle in air and static air contact angle in water. These static contact angles were respectively measured using the sessile drop method and captive bubble method with a contact angle goniometer (Shanghai Zhongchen Digital Equipment Co.). A water droplet or an air bubble was gently deposited onto the surface and the contact angle was recorded once the droplet/bubble profile became stable and the contact line remained stationary. The droplet and bubble volumes were fixed at 1 µL. Advancing and receding contact angles and sliding angles for bubbles were determined using the tilting‐plate method [45]. The sample surface with a 10 µL bubble was gradually tilted until the droplet/bubble started to move, at which point the advancing contact angle, receding contact angle, and sliding angle were recorded. For each surface, at least ten independent measurements were conducted, and the average value was reported.

### 4.6 AFM Force Measurements

Surface interaction and friction force during bubble attachment and sliding on the PDMS network and PDMS CAL surfaces were systematically measured by AFM. All force measurements were performed on three independently prepared substrates, with each experiment repeated at least three different locations on each surface. Representative force curves were presented in the Results section.

### 4.7 Preparation of AFM Bubble Probe

Microbubbles were generated at the bottom of a fluid cell by purging air through an ultrasharp glass pipet into aqueous solution. After immobilizing air bubbles in the fluid cell, a custombuilt tipless rectangular silicon cantilever (400 × 70 × 2 µm^3^) with a hydrophobized gold patch was used to pick up appropriate bubbles (mainly 50–65 µm in radius), and consequently created an AFM bubble probe (Figure S27) [69]. Before this procedure, the fluid cell and cantilever were respectively soaked in OTS solution (10 mM) for different durations to achieve slight and moderate hydrophobic treatment, allowing the temporary immobilization of air bubbles and facilitating bubble pick‐up [69]. The spring constant of AFM bubble probe was calibrated using Hutter‐Bechhoefer method [70].

### 4.8 Surface Interaction Measurements

The bubble probe was placed over the sample surfaces for surface interaction measurements. Specifically, the cantilever was actuated to approach/retract toward the surfaces at a velocity of 1 µm/s, continuing until the interaction forces were detected. The measured deflection of AFM cantilever was represented as force‐time curves by AFM software and fitted using MATLAB, and the SRYL theoretical model is described in the next section. Although the bubble size varied slightly between probes, this variation was taken into account in the fitting procedure. Uncertainties in the fitted parameters primarily arise from calibration accuracy, fitting sensitivity, and local surface heterogeneity. Fitted parameters are therefore reported as best‐fit values within the adopted model, and are used mainly for comparative analysis across different surfaces.

### 4.9 Friction Force Measurements

During the friction force measurements, the sample surfaces were vertically positioned in AFM fluid cell. A bubble probe (mainly 20–25 µm in radius) was created as described above, and then the force balance was set to null to register only the friction force due to bubble movement. Then the bubble was driven to slide along sample surfaces at a nominal speed of 5 µm/s, and the corresponding cantilever deflection was registered and converted into force‐time curves.

### 4.10 Theoretical Models

In this study, a comprehensive theoretical framework was employed to unravel the intricate interaction forces between air bubbles and sample surfaces. The models integrate Reynolds lubrication theory and the augmented Young–Laplace equation (SRYL) by considering the crucial factors such as bubble deformation, pressure, and disjoining pressure arising from surface interactions. By applying this theoretical approach, a deeper understanding of interaction forces underlying bubble attachment can be obtained.

The Reynolds lubrication theory, coupled with immobile boundary conditions, was used to describe the real‐time variation of confined liquid film thickness *h*(*r,t*) between bubbles and sample surfaces, in agreement with previous reports [71, 72]:

(1) $$\frac{\partial h}{\partial t}=\frac{1}{12\mu r}\;\frac{\partial }{\partial r}(rh^{3}\frac{\partial p}{\partial r})$$

Here, *µ* is the dynamic viscosity of solution, *r* is the radical coordinate at which the central point of the bubble is zero, and *p*(*r,t*) indicates the excess hydrodynamic pressure in the liquid film compared to the bulk solution.

The bubble deformation under external forces was described by the extended Young–Laplace equation [20, 72]:

(2) $$\frac{\gamma }{r}\frac{\partial }{\partial r}(r\frac{\partial h}{\partial r})=\frac{2\gamma }{R_{0}}\;-p-\Pi$$

where *γ* is the surface tension of aqueous solution, *r* denotes the bubble radius, and *Π*(*h*(*r,t*)) is the disjoining pressure.

The overall disjoining pressure is contributed by various surface interactions, as previously reported in references [21, 69]. Notably, NaCl solution (500 mm) was diligently employed to effectively screen the electrical double layer (EDL) interaction between air bubbles and sample surfaces. The disjoining pressure of van der Waals (VDW) and hydrophobic (HB) interactions denotes as *Π*_VDW_ and *Π*_HB_, respectively [73]:

(3) $$\Pi_{\mathrm{VDW}}=-\frac{A_{A-W-S}}{6\pi h^{3}\left(r,\;t\right)}$$

(4) $$\Pi_{\mathrm{HB}}=-\frac{\gamma (1-\cos\theta_{W})}{D_{0}}e^{-h/D_{0}}$$

where *A* is Hamaker constant for bubbles interacting with PDMS network/brushes, it is noteworthy that the majority of overall VDW interaction is contributed by PDMS network/brushes due to their thickness exceeding 2 nm, while the impact of silicon substrate is negligible [21]. *D*_0_ is the decay length of hydrophobic interaction and *θ*_W_ donates the surface hydrophobicity.

The total interaction force is mathematically integrated following the Derjaguin approximation principle as follows [69, 74]:

(5) $$F\left(t\right)=2\pi \int_{0}^{\infty }\left[p\left(r,t\right)+\prod \left(h\left(r,t\right)\right)\right]\mathrm{rdr}$$

### 4.11 MD Simulations

The detailed procedures of MD simulations are included in Note S2 in the Supplementary Materials.

## Funding

This work was supported by the National Key R&D Program of China (2023YFC2908900), the Science and Technology Innovation Leading Talent Program of Hunan Province (2024RC1018), the Natural Sciences and Engineering Research Council of Canada (NSERC), the Canada Foundation for Innovation (CFI), and the Canada Research Chairs Program.

## Conflicts of Interest

The authors declare no conflicts of interest.

## Supporting information




**Supporting File 1**: advs75495‐sup‐0001‐SuppMat.docx.

**Supporting File 2**: advs75495‐sup‐0002‐MovieS1‐S6.zip.

## Data Availability

The data that supports the findings of this study are available in the supplementary material of this article.
