# Supplementary material for: Sticky Yet Slippery: Molecular Ordering Reconciles Bubble‐Surface Affinity With Ultralow Friction at the Nanoscale
Source: Adv Sci (Weinh). 2026 May 7;13(41):e75495. doi: 10.1002/advs.75495 (PMC13335574; doi:10.1002/advs.75495)
Supplement: Supplementary file 1 — Supporting File 1: advs75495‐sup‐0001‐SuppMat.docx. [file ADVS-13-e75495-s002.docx]

Supporting Information

Sticky yet slippery: Molecular ordering reconciles bubble-surface affinity with ultralow friction at the nanoscale

Shishuang Zhang, Jiajun Li, Huadong Tian, Zhoujie Wang, Chenyu Qiao, Jingyi Wang, Lei Xie,* and Hongbo Zeng*

Shishuang Zhang, Jiajun Li, Huadong Tian, Zhoujie Wang, Lei Xie

School of Minerals Processing and Bioengineering, Central South University, Changsha, P. R. China

E-mail: lei.xie@csu.edu.cn

Shishuang Zhang, Jiajun Li, Huadong Tian, Lei Xie

Future Resources Interface Science and Intelligent Application Research Center (FRISIARC), Changsha 410083, P. R. China

E-mail: lei.xie@csu.edu.cn

Chenyu Qiao, Hongbo Zeng

Department of Chemical and Materials Engineering, University of Alberta, Edmonton, Canada

E-mail: hongbo.zeng@ualberta.ca

**Jingyi Wang**

College of Chemistry and Chemical Engineering, Southwest Petroleum University, Chengdu, P. R. China

#

Note S1. Force analysis during bubble movement.

After a bubble was deposited on the sample surface, the adhesion force *F*_A_ and the buoyancy *F*_B_ existed, and the resultant force of *F*_A_ and *F*_B_ provided the driving force *F*_1_ for bubble sliding. The frictional resistance *f* was composed of contact angle hysteresis (CAH) and fluid drag, which arose from the contact line movement and water impedance, respectively. The effective forces exerted on the bubble can be calculated by the following equations:^[1,2]^

|  | $\text{F}_{\text{1}}\text{=}\text{ρ}\text{g}\text{V}_{\text{B}}\text{sin}\text{α}$ | (S1) |
| --- | --- | --- |
|  | $\text{f=}\text{f}_{\text{CAH}}\text{+}\text{f}_{\text{D}}$ | (S2) |
|  | $\text{f}_{\text{CAH}}\text{=}\text{k}\text{γw}\text{(cos}\text{θ}_{\text{REC}}\text{–cos}\text{θ}_{\text{ADV}}\text{)}$ | (S3) |
|  | $\text{f}_{\text{D}}\text{=}\frac{\text{1}}{\text{2}}\text{C}_{\text{D}}\text{ρ}\text{v}^{\text{2}}\text{S}$ | (S4) |

where *ρ*, *V*_B_ and *g* are the water density, bubble volume and gravitational acceleration, *α* is the tilting angle of sample surfaces and is fixed at 20°; *k* is a geometric factor that depends on the shape of the drop, $\text{γ}$, *w*, *θ*_REC_ and *θ*_ADV_ donate the surface tension of water, the character width and advancing and receding angle of bubble, respectively; *C*_D_, *v*, and *S* are the coefficient of water drag, bubble velocity, and the front surface cross-sectional area of the bubble, respectively.

Note S2. MD simulations of the arrangement of interfacial water molecules and response to bubble sliding under a constant applied force for different brush layer thickness.

In this study, molecular dynamics simulations were conducted using the LAMMPS software package, developed by Sandia National Laboratories.^[3,4]^ Before delving into the simulation details, it is important to note that PDMS polymer layers of varying thickness were obtained by adjusting the immersion time in the experimental setup. To capture the experimentally observed variation in polymer layer thickness, PDMS brushes with different grafting densities were constructed in the simulations. It should be noted that, to improve computational efficiency and highlight the role of molecular ordering, the simulation parameters were simplified relative to experimental PDMS brush systems, employing comparatively high grafting densities and short chain lengths.^[5,6]^ Although this model does not quantitatively reproduce the experimental structure, it does not alter the obtained central conclusion.

The simulation work in this study was divided into two parts. The first part simulated the states of interfacial water molecules on PDMS grafted surfaces with different grafting densities, with a focus on the arrangement and orientation of hydroxyl groups in the water molecules near the PDMS brushes. The second part primarily explored how air bubbles (composed mainly of N_2_ and O_2_) slide along PDMS brushes of varying thickness underwater.

The simulations were performed using the same material design as the experiments, where single-crystal silicon (100) was used as the substrate for grafting polymer brushes. Initially, OH groups were introduced onto the silicon terminal surface to simulate the hydroxylation reaction during the grafting of PDMS polymer chains onto the surface. Subsequently, Si-O-Si bonds were formed through a dehydration reaction between the OH groups of PDMS polymer chains and OH groups on the silicon substrate. Three different surface coverages (30%, 50%, and 70%) were set using a surface doping Perl script in the Materials Studio software to simulate low, medium, and high PDMS brush thickness experimental conditions.

Unstable conformations or locally energetic configurations may exist in PDMS brush layers, since PDMS chains were grafted directly onto the hydroxylated silicon substrate by artificial design. To obtain simulated PDMS polymer chains similar to the experimental case, the structure was optimized using compression pretreatment. Specifically, external compressive stress was gradually applied through carbon plates to eliminate voids and unreasonable tensions using a custom Perl script, which resulted in a compact chain arrangement representing the actual physical state. A 500 ps NVT ensemble simulation was performed on PDMS-modified substrate/vacuum system to relax the PDMS molecules on Si substrate. In the simulation, periodic boundary conditions were applied in *x* and *y* directions, while *z* direction was set to non-periodic. The system temperature was maintained at 300 K using a Nosé-Hoover thermostat with a damping coefficient of 100 ps.

The model sizes of PDMS grafted substrate system described above were different in the following two simulation parts, and other settings remained unchanged. The configuration of mixed gases within air bubble was emphasized. According to the study by Lei et al., the gas density within smaller bubbles may be higher at the same volume.^[7]^ Through molecular dynamics simulations, they found that nanobubbles with radius of 14 Å, 18 Å, and 22 Å had internal pressures of 792 atm, 647 atm, and 518 atm, respectively. Fang et al. reported that the density of N_2_ within bubbles could reach up to 260 kg/m^3^.^[8]^ Furthermore, the stability of bubbles was enhanced on graphite surfaces, potentially leading to an increased internal gas density. Chen et al. demonstrated that surface bubbles could remain stable for over 160 ns in the absence of surface pinning points, and the gas layer on the hydrophobic substrate was crucial for the stability of nanobubbles.^[9]^ Using molecular dynamics simulations, Maheshwari et al. confirmed that contact line pinning and gas supersaturation were crucial elements for bubble stability.^[10]^ In their studies, the interactions between particles were described using the Lennard-Jones potential. In this study, PDMS grafted surfaces serve as a hydrophobic substrate, where the stable presence of mixed gases is a prerequisite for conducting bubble sliding experiments. Sakari Lepikko et al. investigated the sliding behavior of droplets on solid surfaces with varying coverage of self-assembled monolayers (SAMs), which revealed the influence of SAM coverage on the frictional force on water droplets.^[11]^ Their findings provide a simulation framework in this study.

Based on the aforementioned studies, the mixed gases were introduced in this simulation according to the mass ratio of N_2_ and O_2_ in air. The density and radius of bubble hemisphere were respectively set to 0.6 g/cm^3^ and 15 Å. Based on number density, 18 O_2_ molecules and 70 N_2_ molecules were calculated within the bubble. The bubble configuration is only relevant to the second part of the simulation.

Water molecules were arranged in a simple cubic lattice with a lattice constant of 3.0 Å using the TIP3P model. Here, the number of water molecules remained unchanged, allowing PDMS brush thickness to be the only variability. The constructed hemispherical bubble, defined using the region command, was placed within the water molecule layer. A 3 Å interval between the bubble and the water layer was maintained to facilitate the subsequent equilibration.

In the simulation, the Lennard-Jones (LJ) potential between different atoms was calculated using the geometric mean mixing rule. The cutoff radius for van der Waals interaction and long-range electrostatic interaction was set to 8 Å and 10 Å, respectively. The coulombic forces were computed using the PPPM method with a relative precision of 1×10^–4^.^[12]^ Temperature was controlled using the Nose-Hoover method with a damping coefficient of 100 fs.^[13]^ Energy minimization was performed using the conjugate gradient method. Periodic boundary conditions were applied in the *x*, *y*, and *z* directions, and the time step was set to 1 fs. The interactions between all atoms were modeled using the Consistent Valence Force-Field (CVFF), where Si crystal was set to be fixed in the simulation.^[14]^ The CVFF force field parameters were assigned by the Forcite tool in BIOVIA Materials Studio software, and the relevant formulas are as follows:

|  | $\begin{aligned} \text{E}\text{=}\sum_{\text{bond}} \text{ }\text{K}_{\text{b}}\text{(}\text{b}\text{–}\text{b}_{\text{0}}\text{)}^{\text{2}}\text{+}\sum_{\text{angle}} \text{ }\text{K}_{\text{θ}}\text{(}\text{θ}\text{–}\text{θ}_{\text{0}}\text{)}^{\text{2}}\text{+}\sum_{\text{torsion}} \text{ }\text{K}_{\text{φ}}\left[ \text{1+}\cos\text{(}\text{n}\text{φ}{\text{–}\text{φ}}_{\text{0}}\text{)}\text{(}\text{nφ}{\text{–}\text{φ}}_{\text{0}}\text{)} \right] \\ \sum_{\text{out-of-plane}} \text{ }\text{K}_{\text{χ}}\left[ \text{1+}\cos\text{(}\text{n}\text{χ}\text{–}\text{χ}_{\text{0}}\text{)} \right]\text{+}\sum_{\text{L-J}} \text{ }\left[ \left( \frac{\text{A}_{\text{ij}}}{\text{r}_{\text{ij}}} \right)^{\text{12}}\text{–}\left( \frac{\text{B}_{\text{ij}}}{\text{r}_{\text{ij}}} \right)^{\text{6}} \right]\text{+}\sum_{\text{Coulomb}} \text{ }\frac{\text{C}\text{q}_{\text{i}}\text{q}_{\text{j}}}{\text{ε}\text{r}_{\text{ij}}} \end{aligned}$ | (S5) |
| --- | --- | --- |

*K*_b_, *K*_θ_, *K*_φ_, and *K*_χ_ are the force constants; *b*_0_, *θ*_0_, *φ*_0_, and *χ*_0_ are the equilibrium bond lengths, bond angles, dihedral angles, and out-of-plane angles, respectively; *b*, *θ*, *φ*, and *χ* are the bond lengths, bond angles, dihedral angles, and out-of-plane angles; *n* is the periodicity parameter; *A_ij_* and *B_ij_* are the square roots of the products of *A_i_* with *A_j_* and *B_i_* with *B_j_*, respectively; *C* and *ε* are the energy conversion constants and the tolerance coefficients; *r_ij_* is the distance between atoms *i* and *j*, which are charged *q_i_* and *q_j_*, respectively. In CVFF, partial charges were estimated using the bond increment method. For atom *i*, the total charge is calculated from the sum of *j* contributions as given by the formula.

|  | $\text{q}_{\text{i}}\text{=}\sum_{\text{j}} \text{δ}_{\text{ij}}$ | (S6) |
| --- | --- | --- |

where *j* represents all atoms directly bonded to atom *i;* the bond increment *δ*_ij_ represents the partial charge of atom *j* on atom *i*, and this method assigns equal magnitude but opposite sign *δ*_ij_ to each bonded pair of atoms *i* and *j*.

In the first part of the simulation, the arrangement of hydroxyl groups on water molecules near PDMS surface was present by using a Nosé-Hoover thermostat to run the system at 300 K in the NVT ensemble for 500 ps. In the second part of the simulation, the bubble sliding behavior on PDMS grafted surfaces was studied with a duration of 100 ps. The system was first equilibrated using a Nosé-Hoover thermostat at 300 K in the NVT ensemble for 100 ps. Subsequently, a constant force was applied in the *x* direction to O_2_ and N_2_ molecules within the NVT ensemble, which was proportional to the gas molecule mass to ensure consistent acceleration of O_2_ and N_2_. The calculated forces on O_2_ and N_2_ molecules were approximately 0.0427 and 0.0373 kcal/mol/Å, respectively. To maintain the initial bubble pressure, a repulsive virtual wall with a 12-6 potential was added above the water layer to limit the bubble expansion. Finally, post-processing analysis of atomic trajectories was visualized using the OVITO software.

In addition to the bubble sliding simulations, bubble detachment from PDMS-grafted surfaces was also investigated. The simulation setup was identical to that used in the sliding simulations, including the system configuration, force field parameters, and thermodynamic ensemble. To evaluate the resistance of the PDMS brush layers to bubble removal, an external driving force was applied to the gas molecules along the positive z direction, i.e., normal to the substrate surface, promoting upward bubble motion. This external bias was introduced solely as a controlled perturbation to initiate bubble detachment and does not aim to reproduce spontaneous, thermodynamically driven bubble detachment observed in experiments. Instead, this approach provides a comparative probe of the relative interfacial stability of bubbles on PDMS brush layers with different grafting densities.


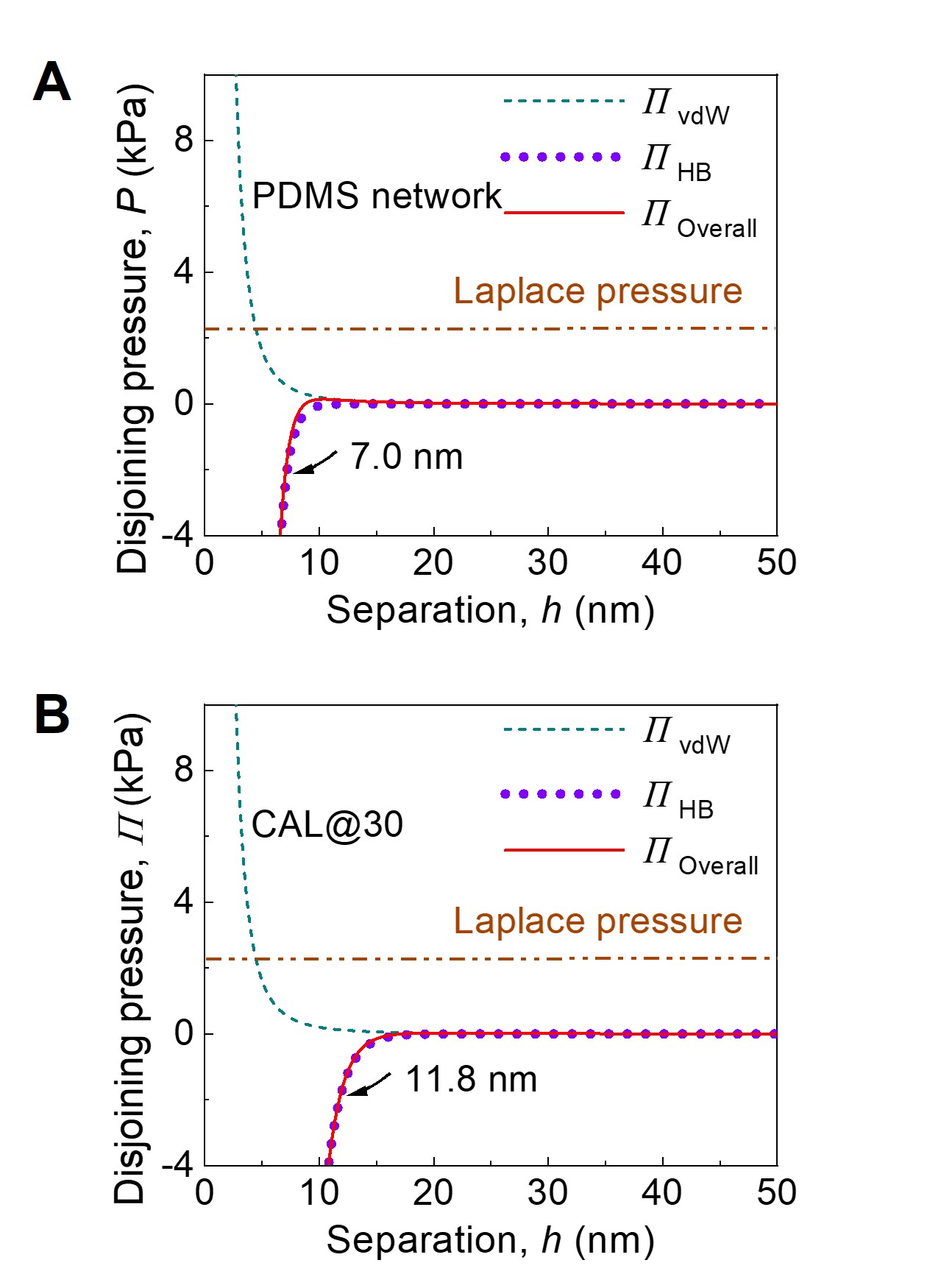


**Figure S1.** Theoretically reproduced disjoining pressure-separation profiles for the involved surface interactions between an air bubble and (A) PDMS network and (B) CAL@30, respectively.


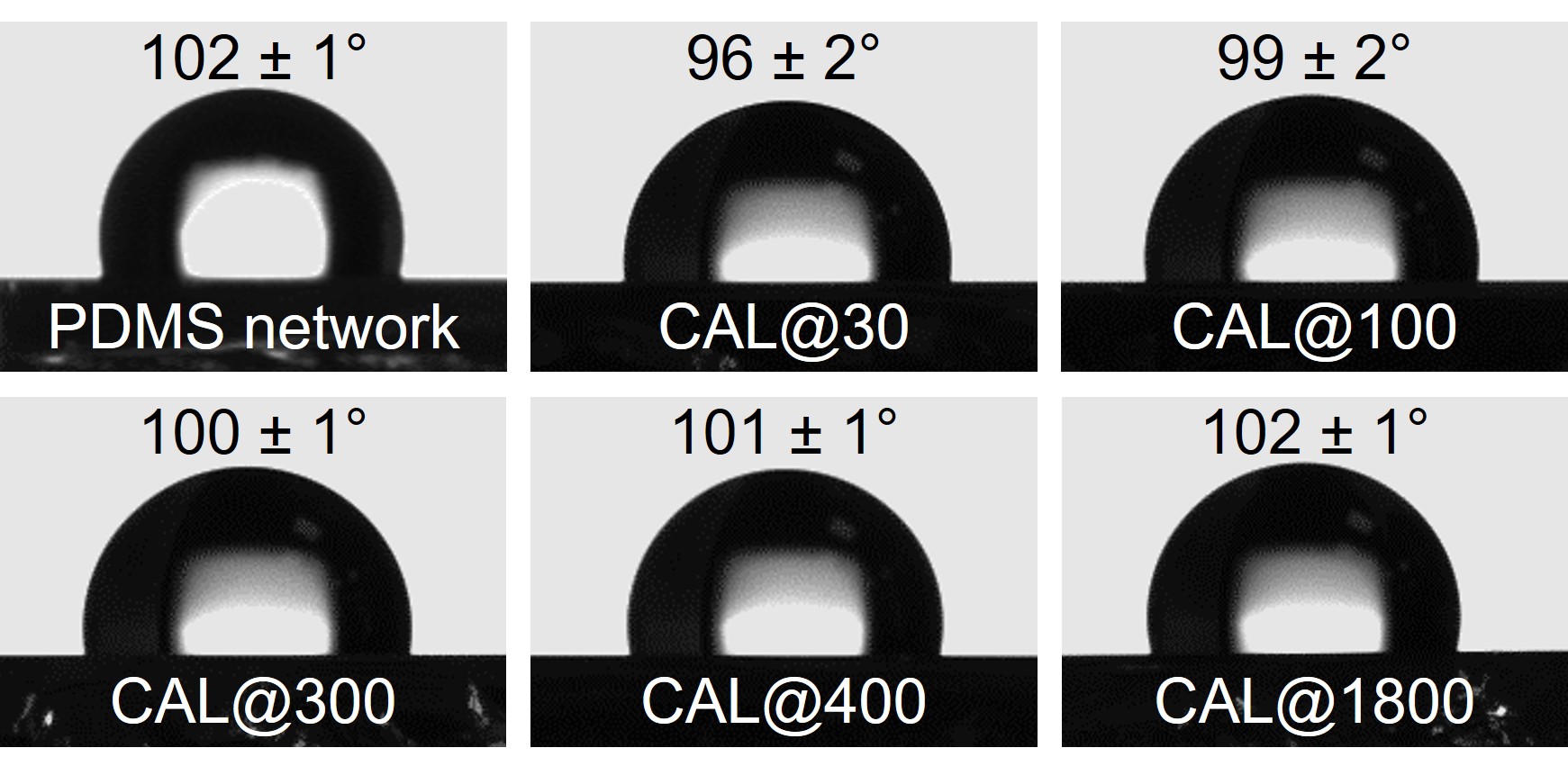


**Figure S2.** Images of water droplets in air on PDMS network and PDMS CAL surfaces, respectively. The error values in the plates denotes the standard deviations of 10 individual contact angle measurements.


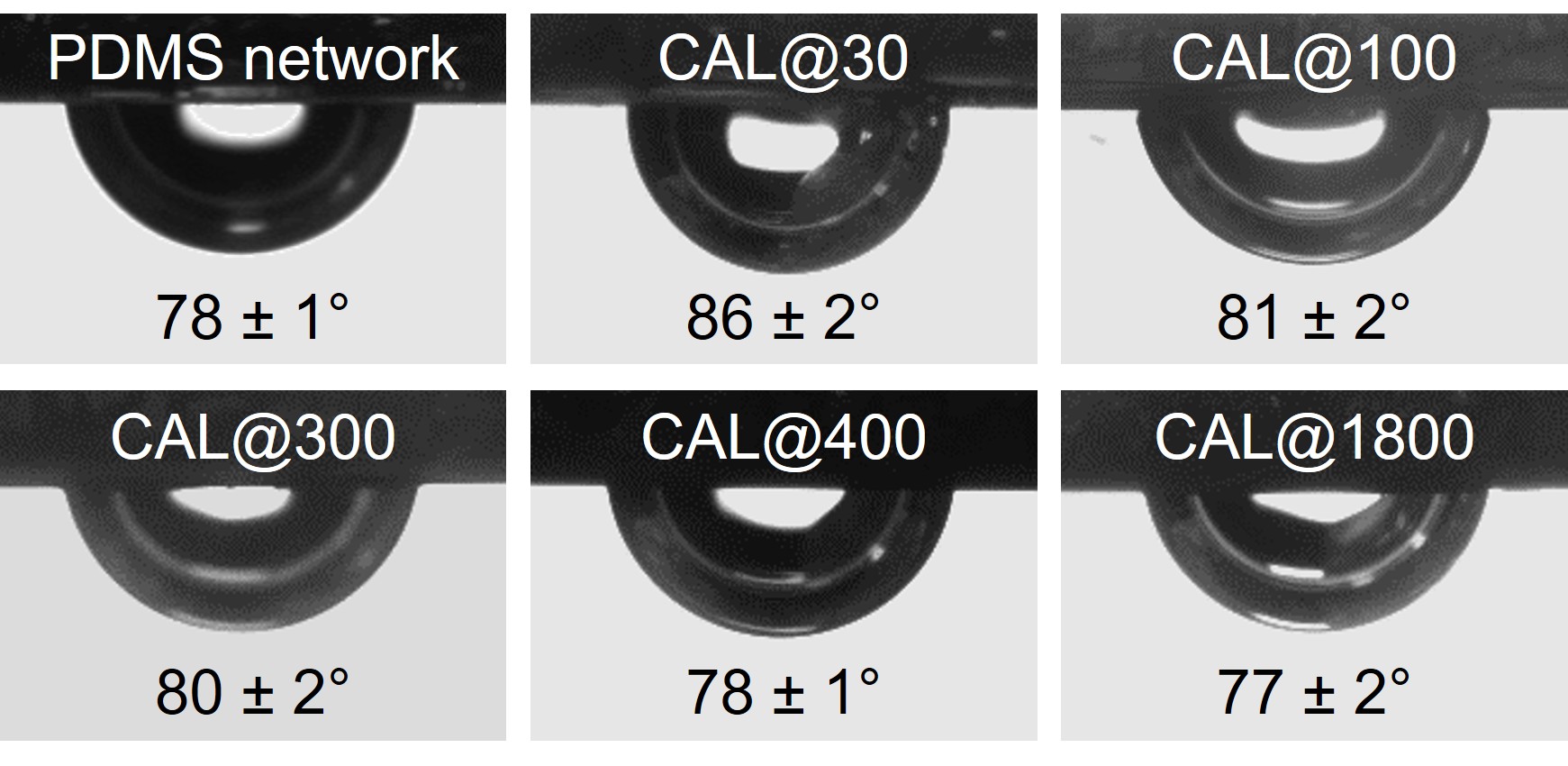


**Figure S3.** Images of air bubbles in water on PDMS network and PDMS CAL surfaces, respectively. The error values in the plates denotes the standard deviations of 10 individual contact angle measurements.


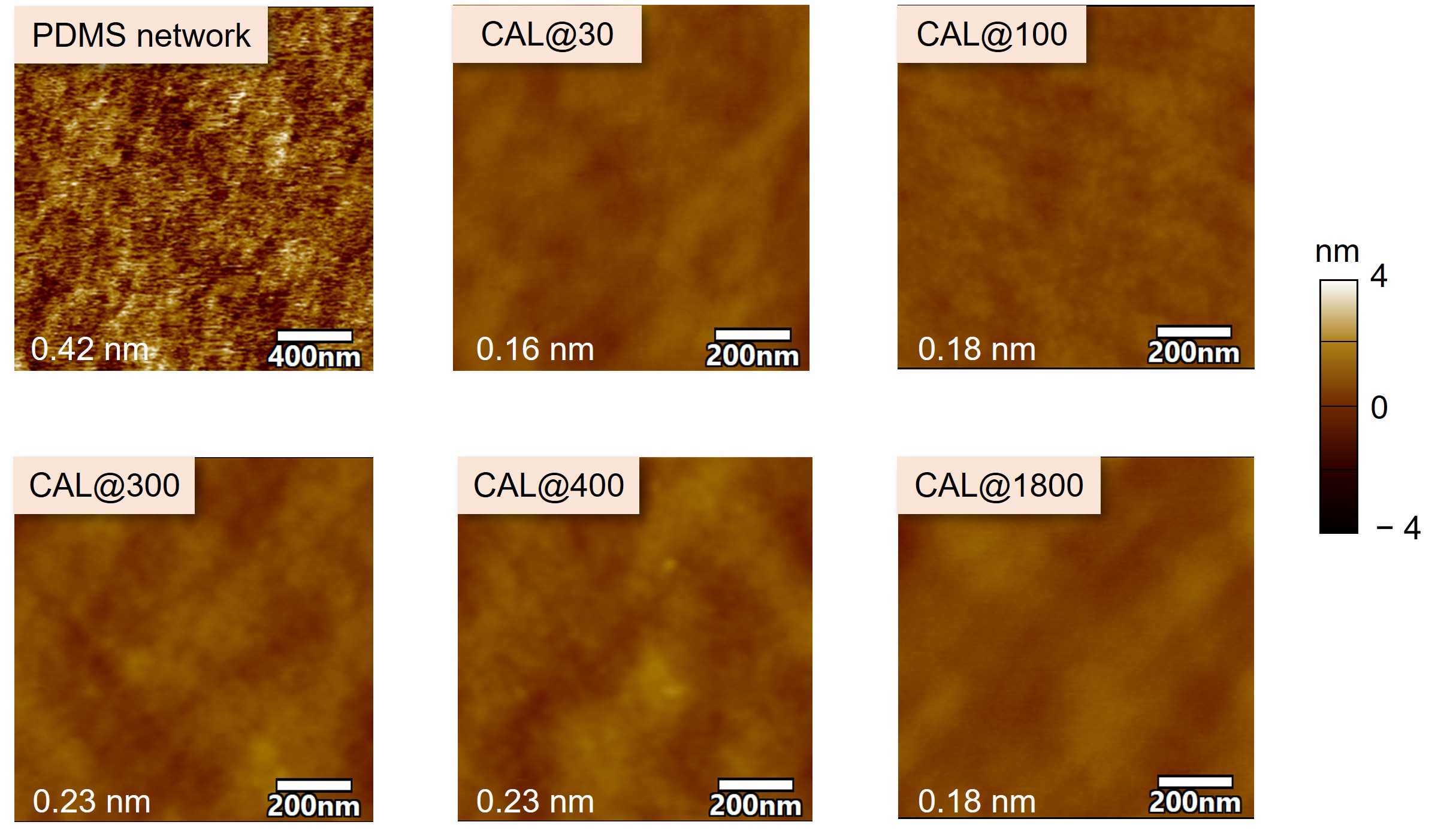


**Figure S4.** AFM morphological images of the prepared PDMS network (2 μm × 2 μm) and PDMS CAL surfaces (1 μm × 1 μm).


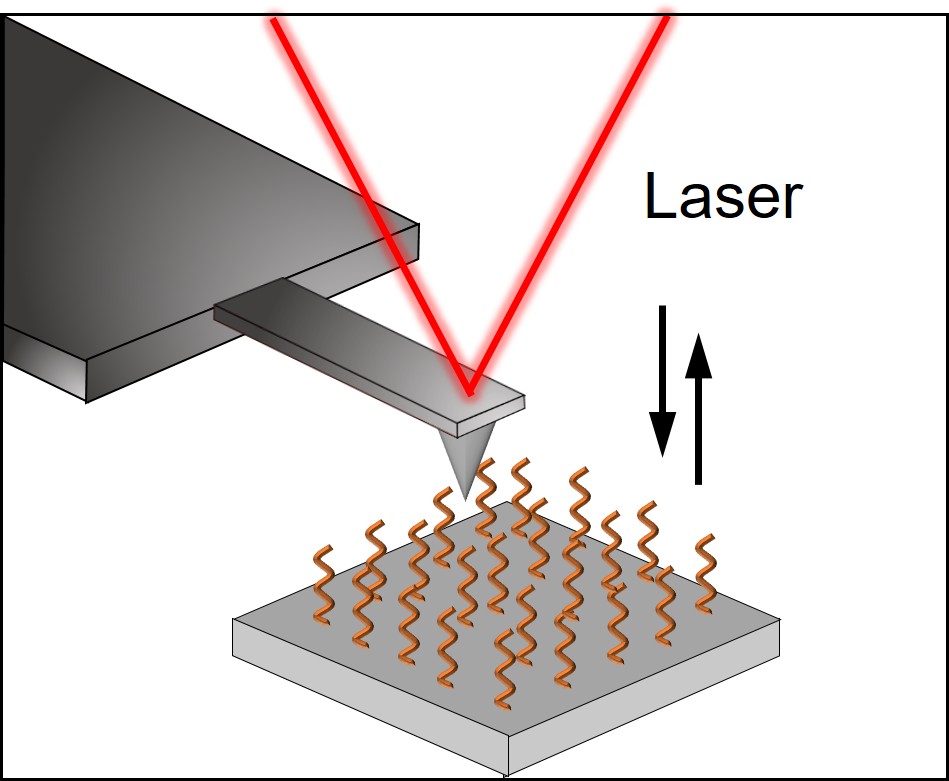


**Figure S5.** AFM force measurement for characterizing PDMS brush thicknesses.


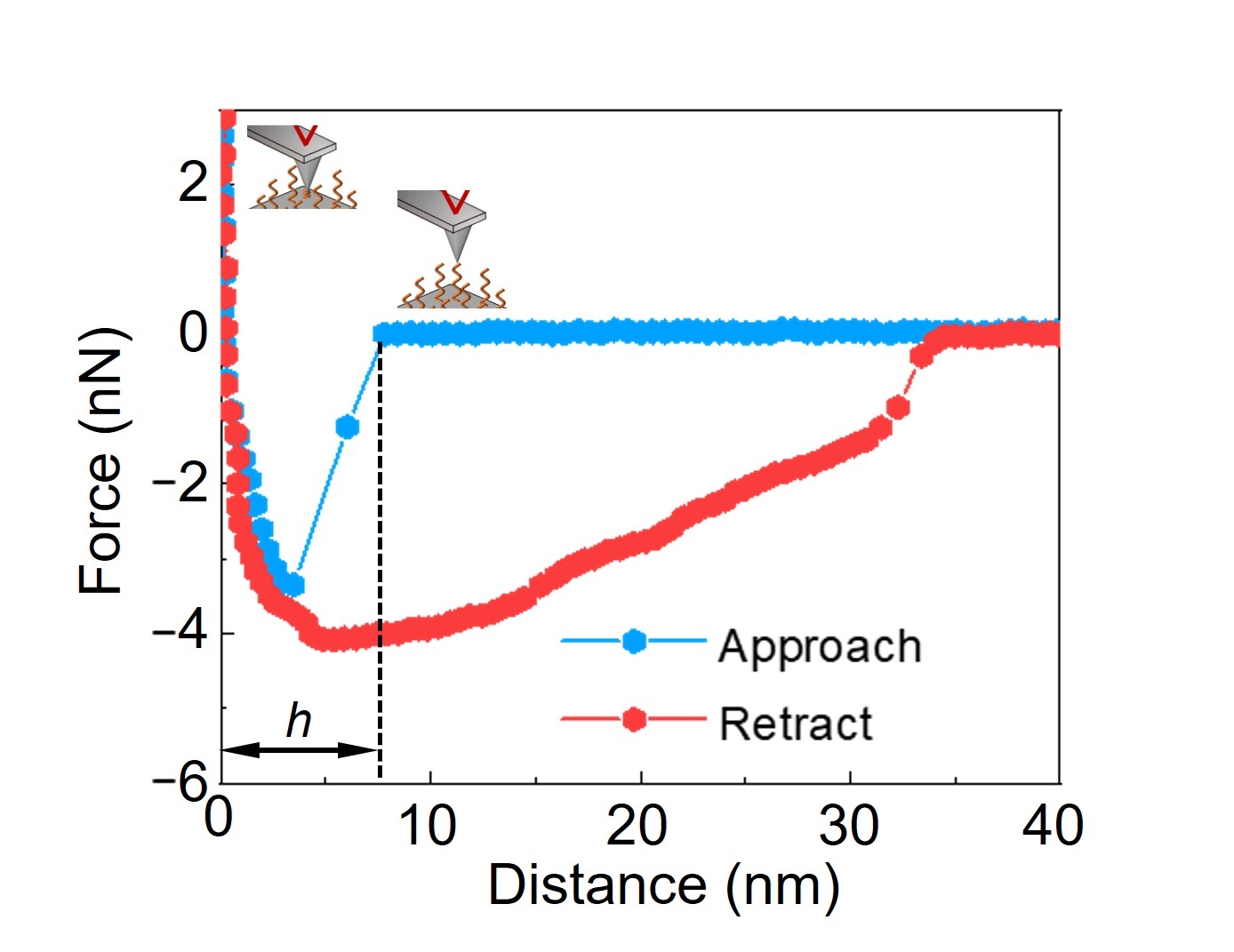


**Figure S6.** A typical force curve for extracting brush thickness *h*.


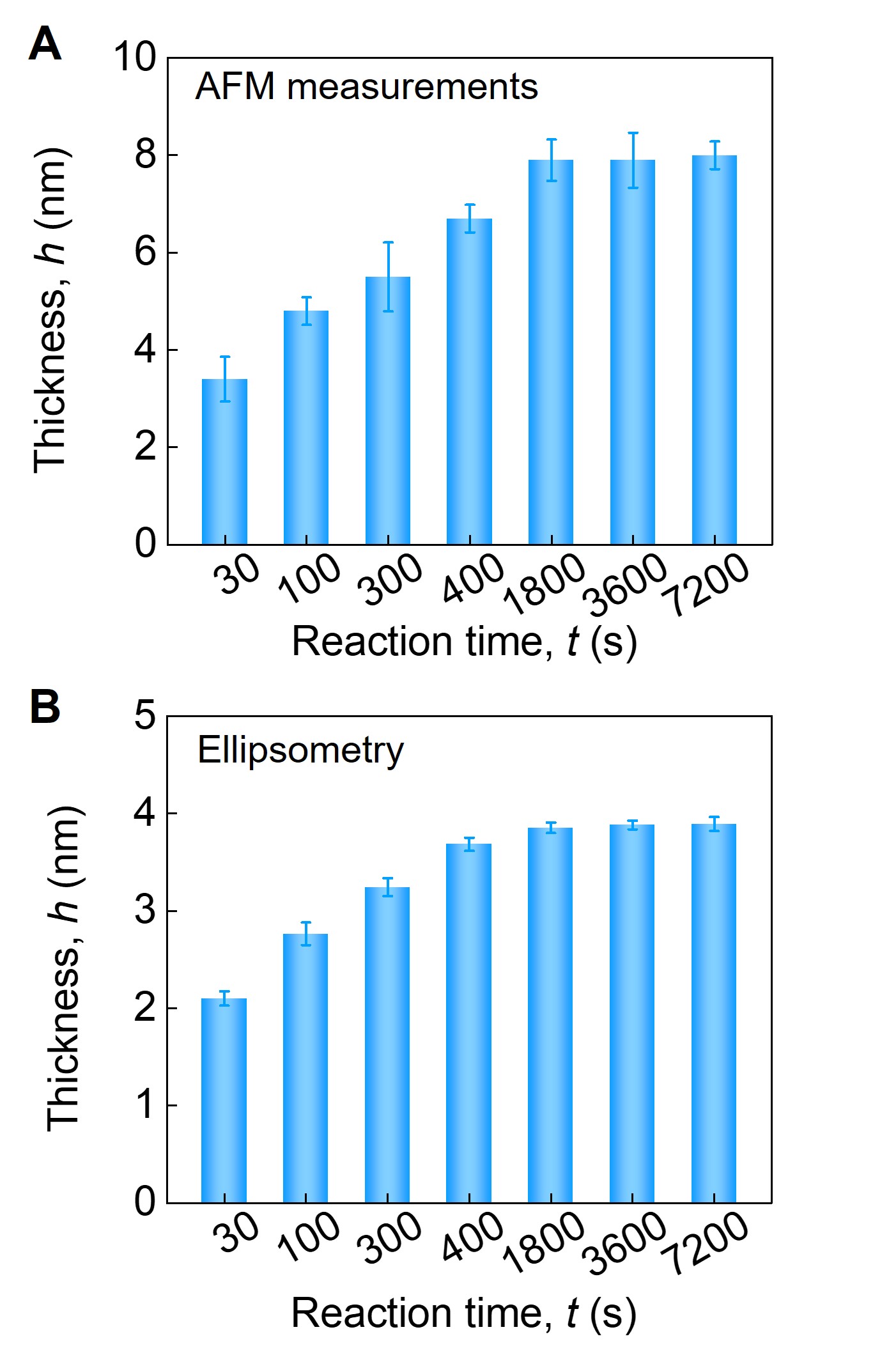


**Figure S7.** Growth kinetics of PDMS brushes on a silicon substrate determined by (A) AFM and (B) ellipsometry measurements, respectively. It should be noted that AFM force measurements in air tend to over-state the thickness due to the stretch deformation of PDMS molecules and possible presence of thin water layers. Given the intrinsic uncertainty associated with force-based thickness extraction, ellipsometry-derived thickness values are therefore reported in the main text.


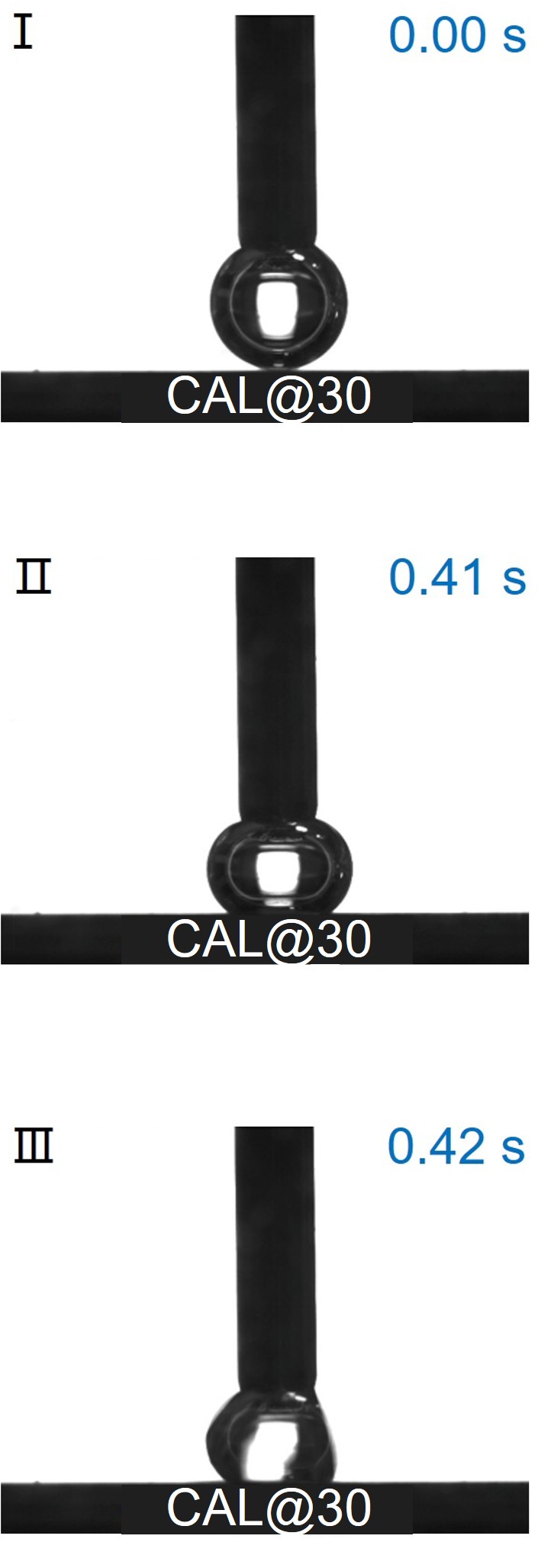


**Figure S8.** Intercepted frames of bubbles from attachment to spreading on CAL@30 during the approaching process. Here, I represents the moment when the bubble first contacts the surface, II depicts the moment just before spreading, and III shows the bubble just spreading over the surface.


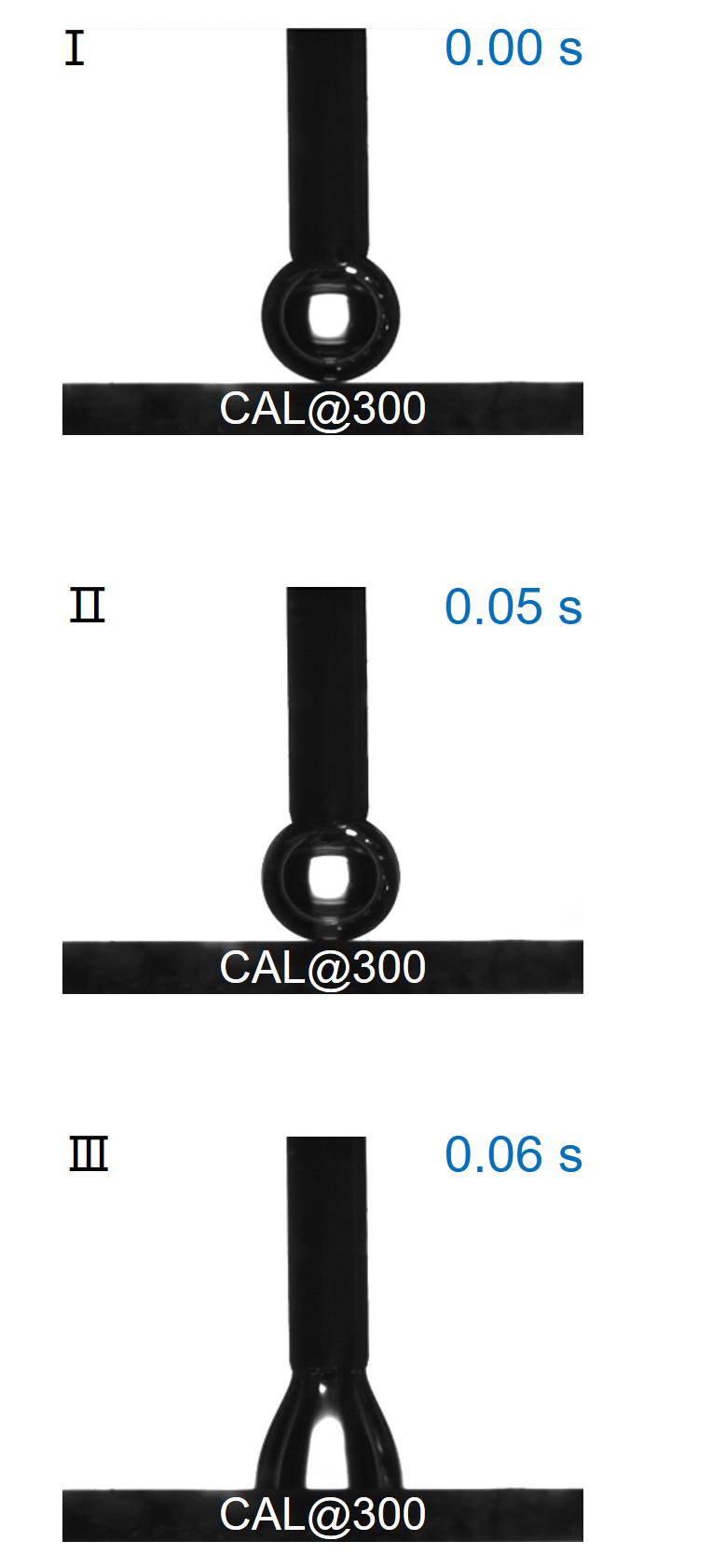


**Figure S9.** Intercepted frames of bubbles from attachment to spreading on CAL@300 during the approaching process. Here, I represents the moment when the bubble first contacts the surface, II depicts the moment just before spreading, and III shows the bubble just spreading over the surface.


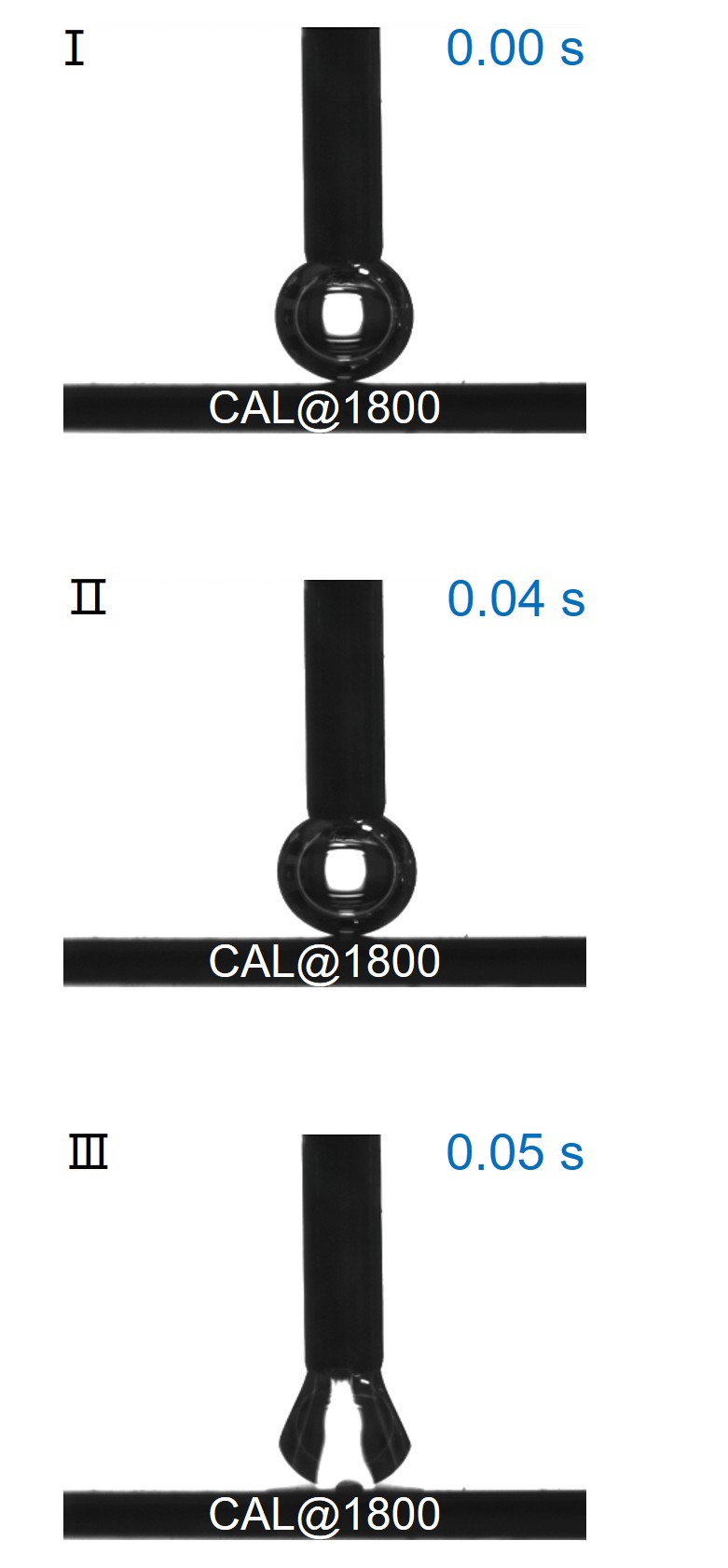


**Figure S10.** Intercepted frames of bubbles from attachment to spreading on CAL@1800 during the approaching process. Here, I represents the moment when the bubble first contacts the surface, II depicts the moment just before spreading, and III shows the bubble just spreading over the surface.


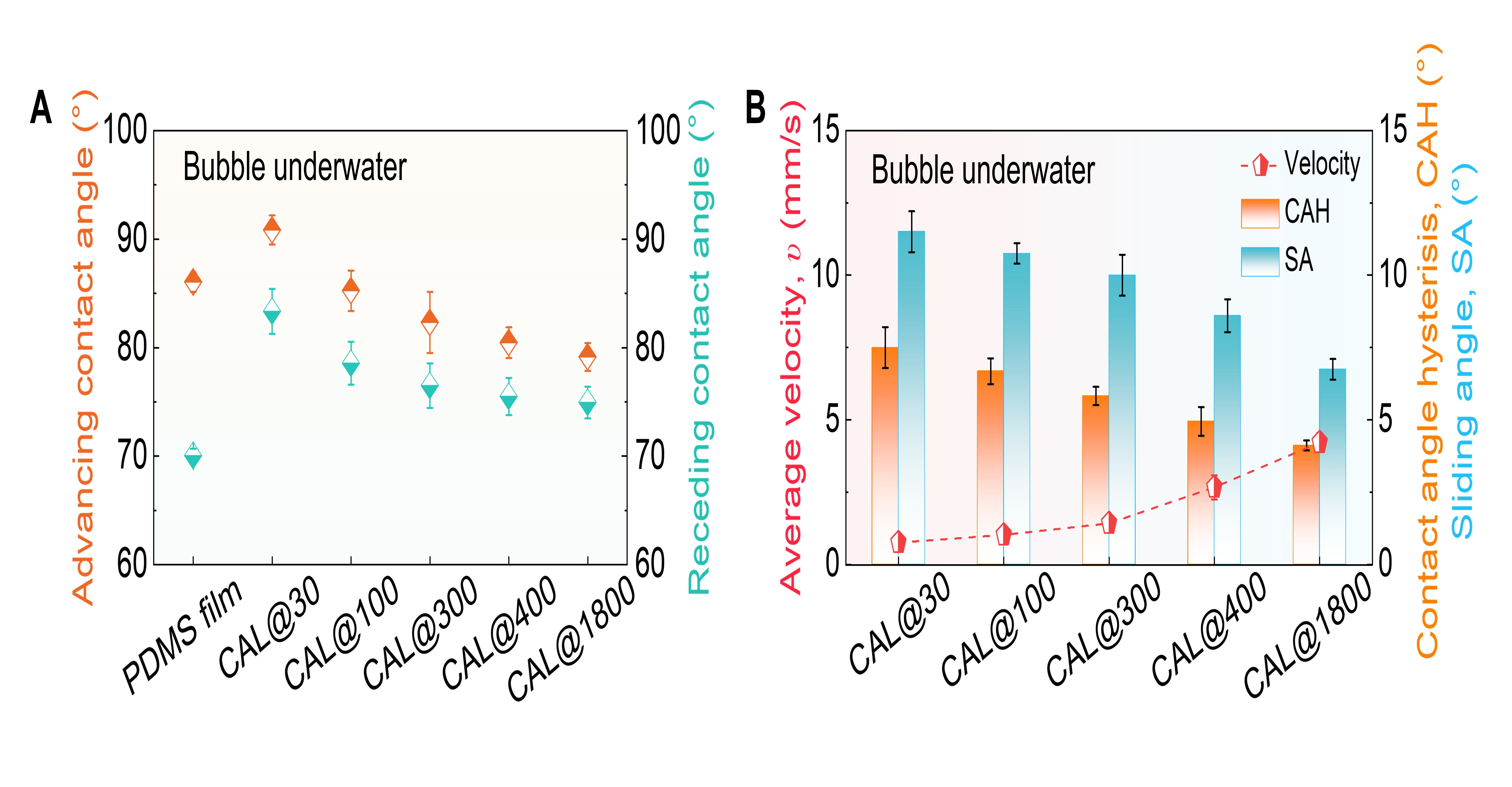


**Figure S11.** (A) Advancing and receding contact angles for bubbles on PDMS film (86° *vs* 70°), CAL@30 (93° *vs* 86°), CAL@100 (87° *vs* 81°), CAL@300 (92° *vs* 86°), CAL@400 (88° *vs* 83°) and CAL@1800 (89° *vs* 85°). (B) Corresponding contact angle hysteresis (CAH), together with the measured sliding angle (SA) and sliding velocity of bubbles on CAL@30, CAL@100, CAL@300, CAL@400, and CAL@1800.


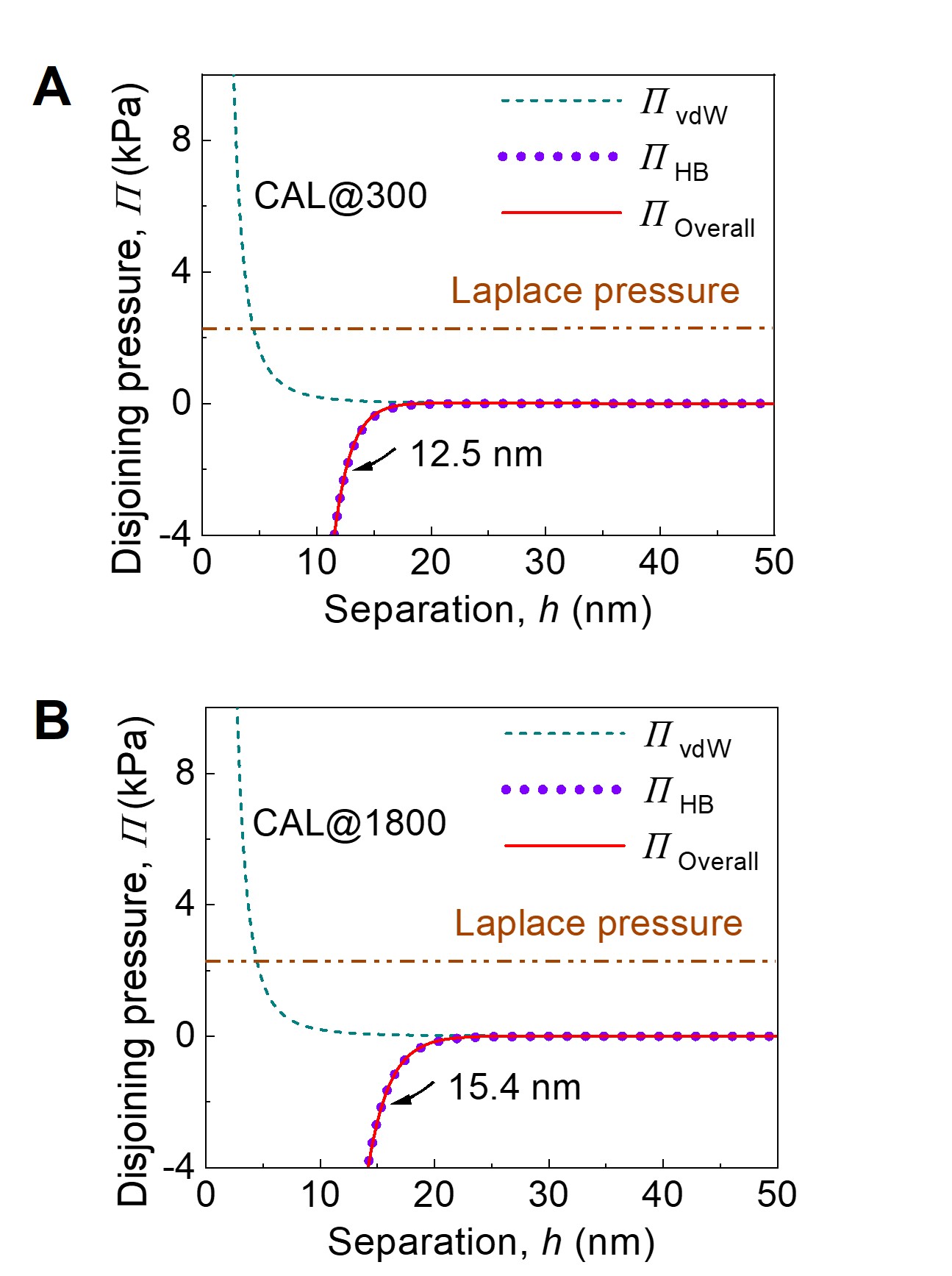


**Figure S12.** Theoretically reproduced disjoining pressure-separation profiles for the involved surface interactions between an air bubble and (A) CAL@300 and (B) CAL@1800, respectively.


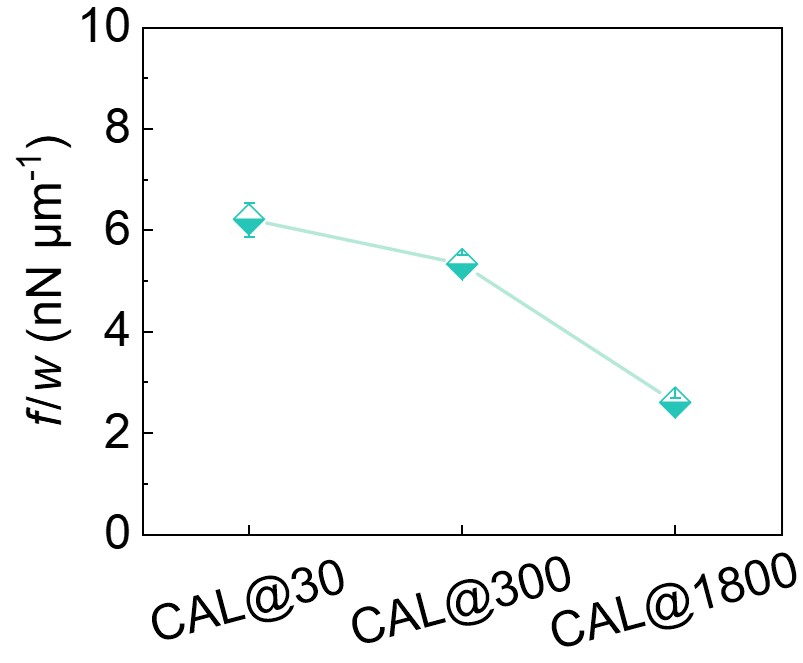


**Figure S13.** Normalized kinetic friction forces *f* */* *w* for bubble sliding on CAL@30, CAL@300 and CAL@1800.


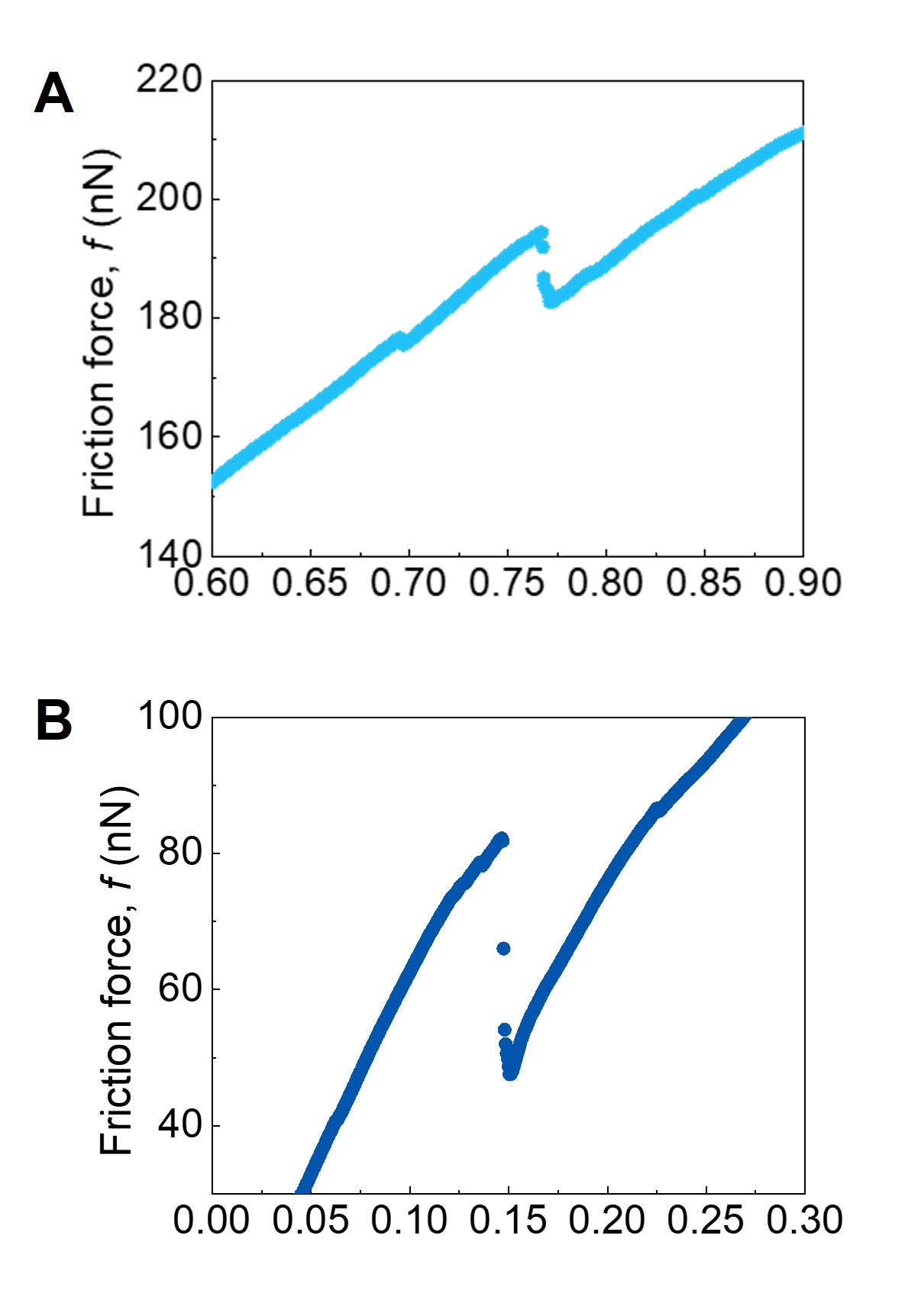


**Figure S14.** Magnified view of friction force during bubble sliding along (A) CAL@30 and (B) CAL@300. Here, the stick-slip motion is evident at 0.75 s and 0.16 s, respectively.


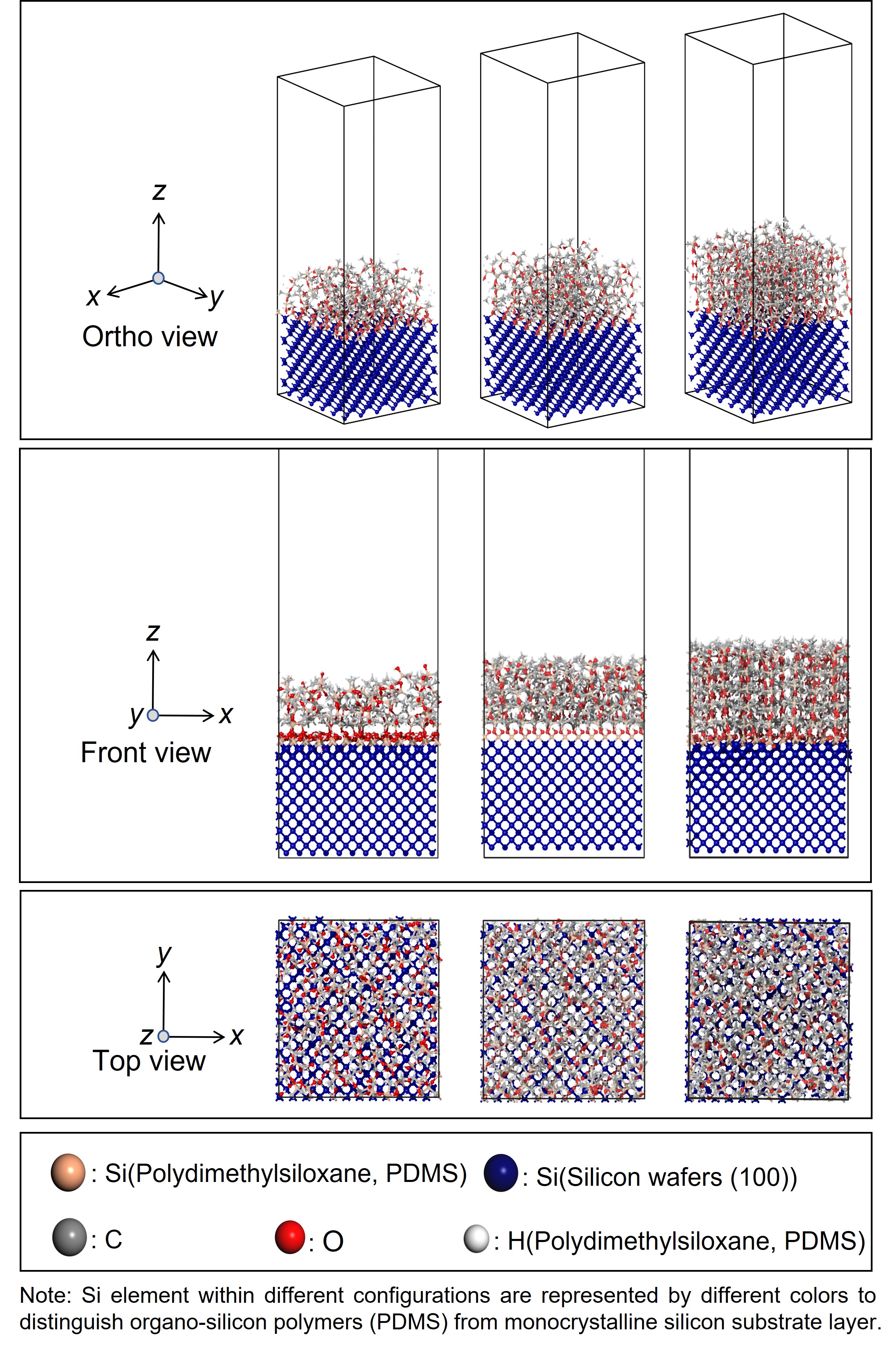


**Figure S15.** The view of a silicon substrate modified with PDMS polymers in the first part simulation.


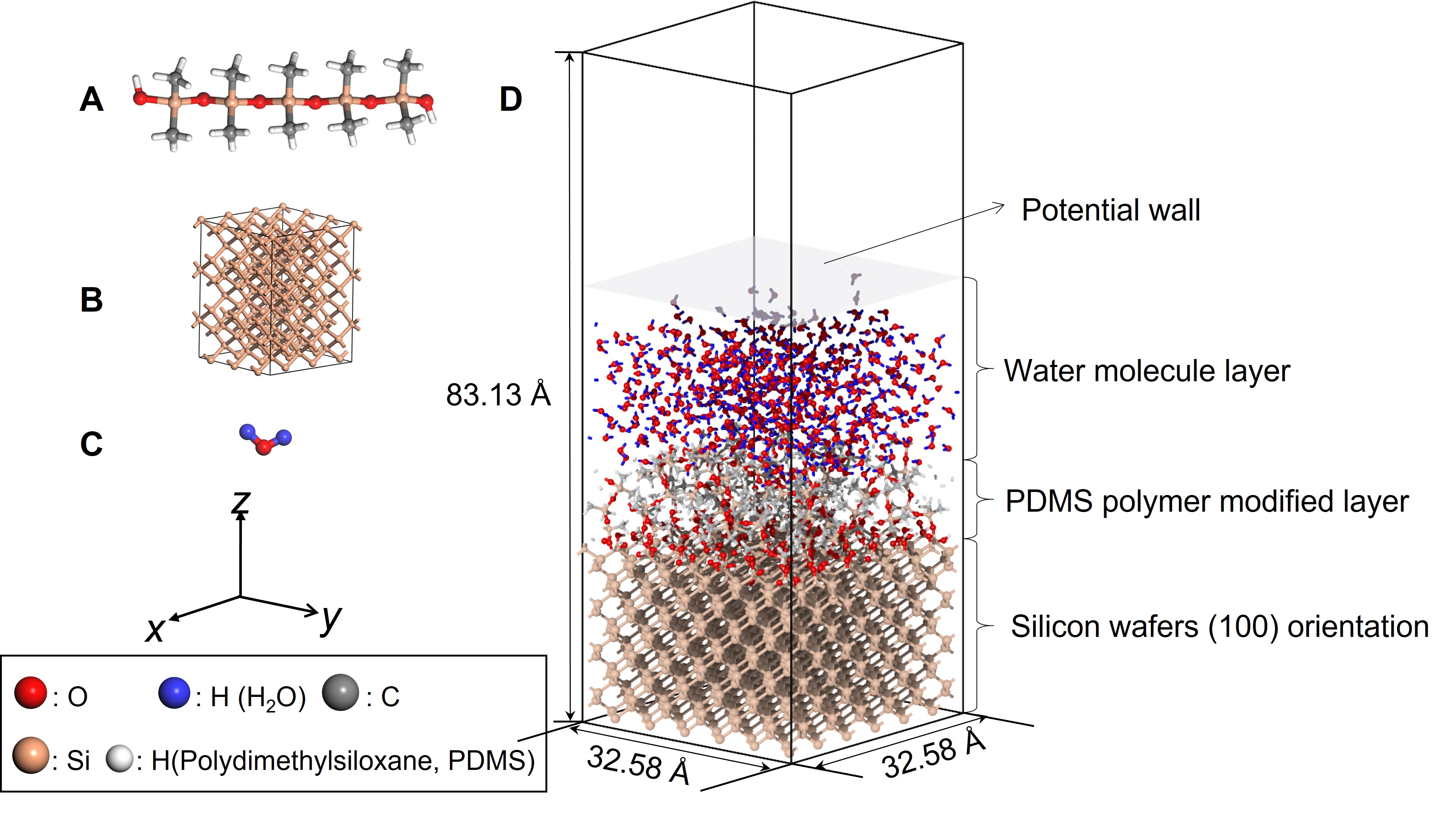


**Figure S16.** Schematic illustration of the simulated components. (**A**) PDMS molecular structure. (**B**) Unit cell of single-crystal silicon (100). (**C**) Water molecule model. (**D**) Overhead view of PDMS grafted silicon substrate/water molecule layer system.


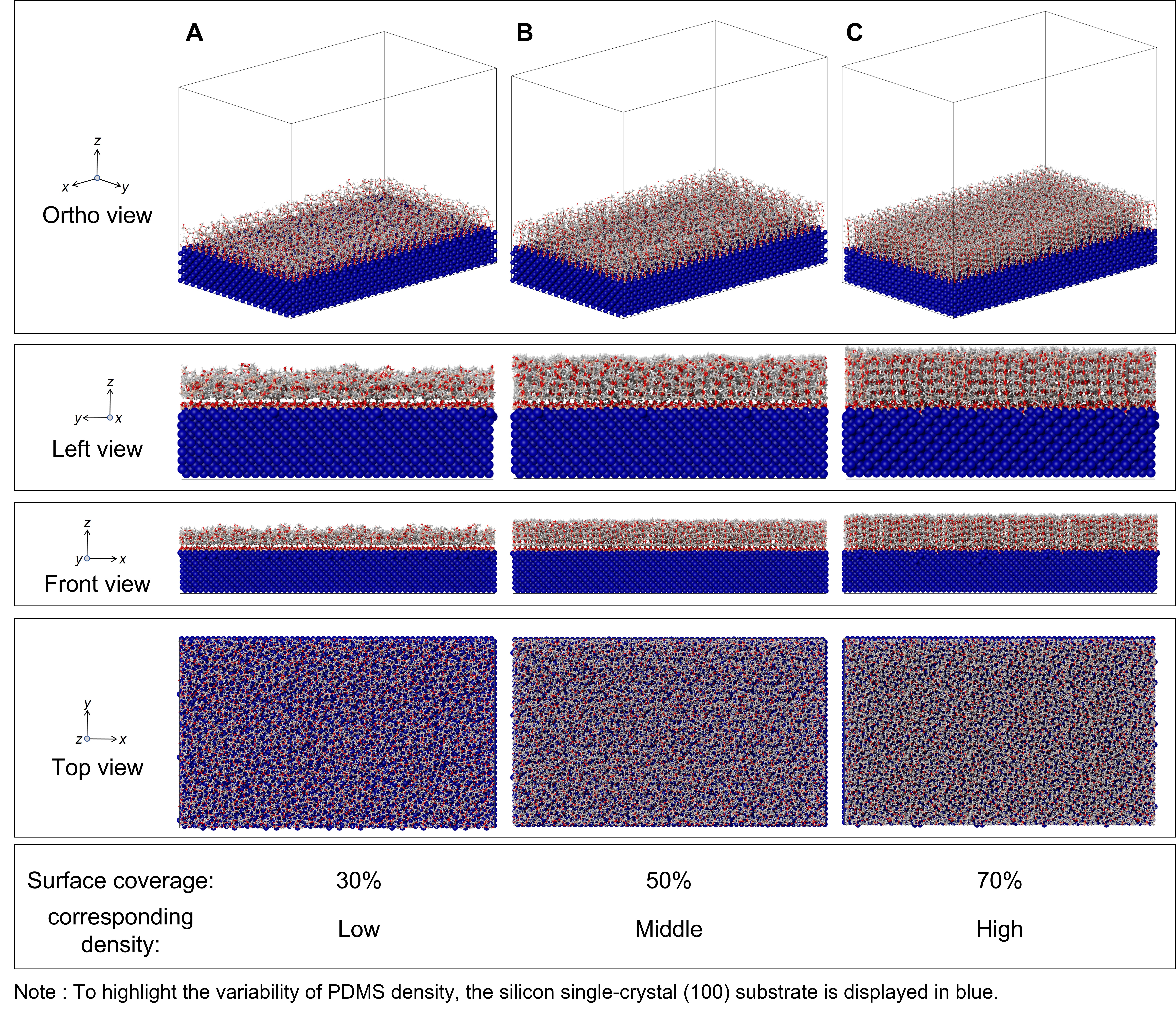


**Figure S17.** Three-view projections of a PDMS-modified silicon substrate in the second part simulation. (**A**) System with 30% PDMS grafting density. (**B**) System with 50% PDMS grafting density. (**C**) System with 70% PDMS grafting density.


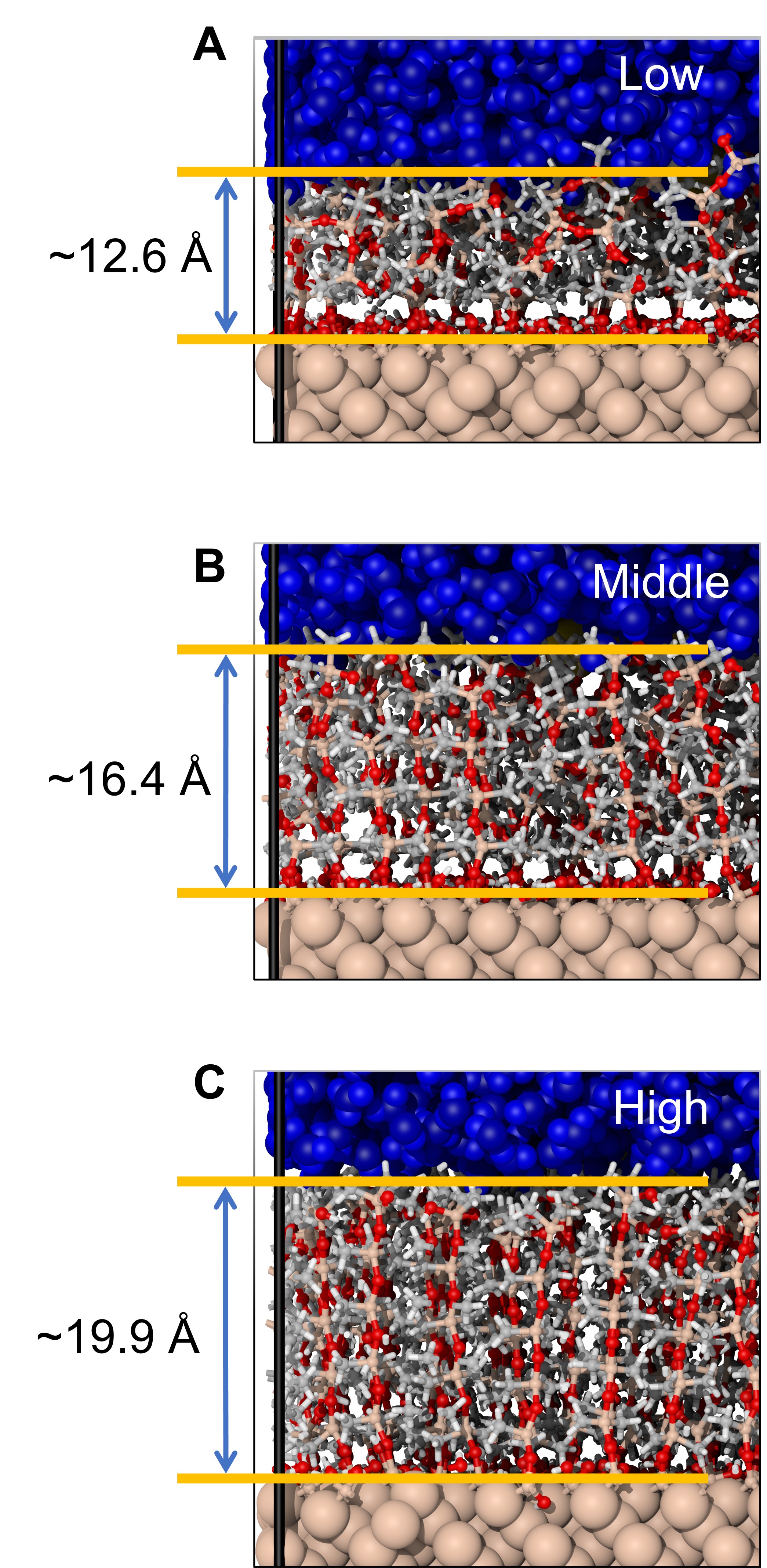


**Figure S18.** Polymer brush layers of varying thickness formed by different PDMS grafting density. (A) System with 30% PDMS grafting density. (B) System with 50% PDMS grafting density. (C) System with 70% PDMS grafting density.


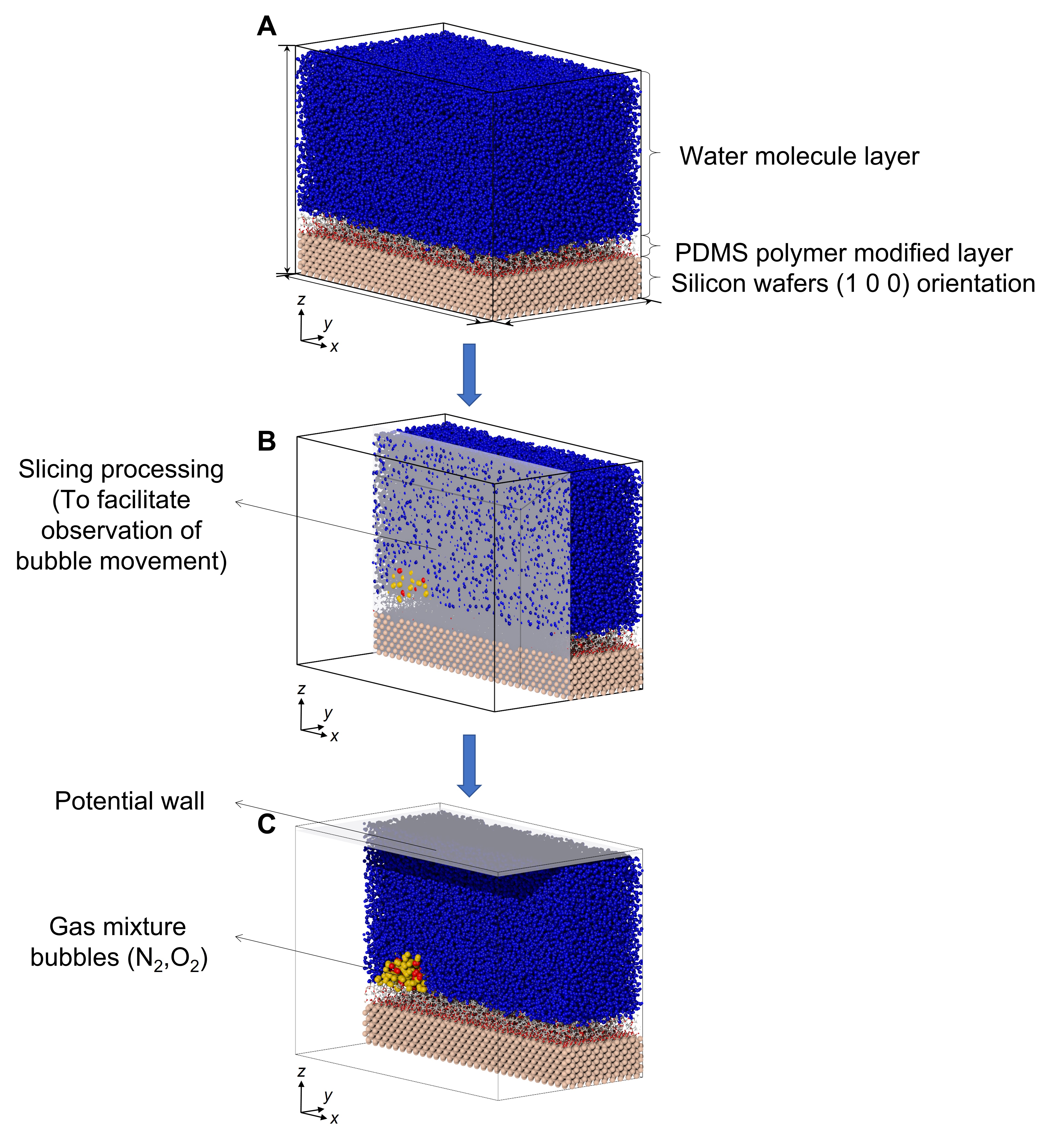


**Figure S19.** Slicing to facilitate observing the bubble sliding behavior within water molecule layer. To prevent unphysical bubble over-expansion and artificial pressure decay within the finite simulation box, a repulsive 12-6 virtual wall was implemented at the upper boundary. This wall provides necessary physical confinement to maintain a stable thermodynamic environment and constant bubble pressure. Notably, the wall is positioned sufficiently far from the solid surface so that it only becomes active when the bubble reaches the boundary; thus, the bubble sliding and detachment processes remain entirely unaffected by the wall potential.


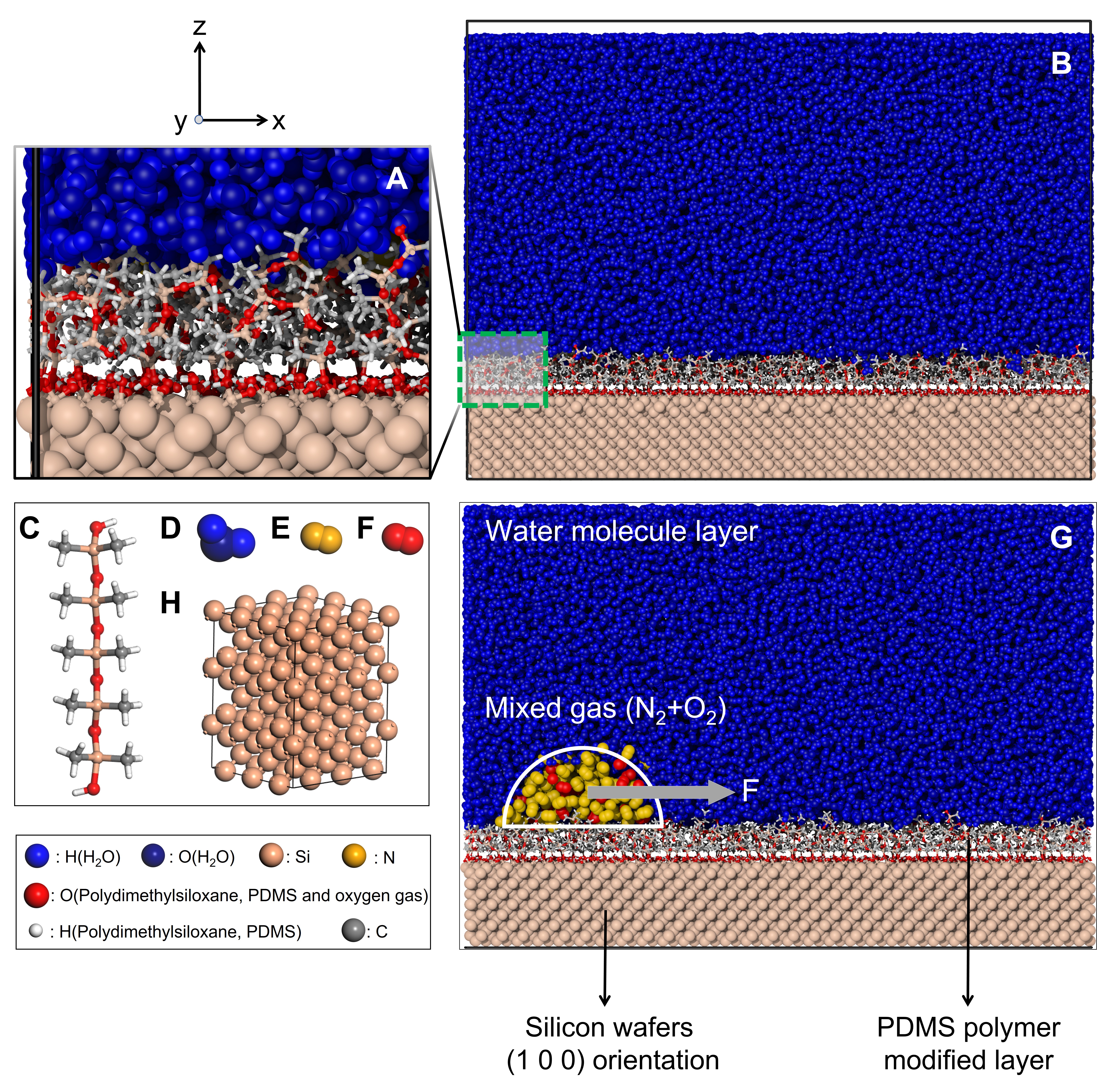


**Figure S20.** (A) Enlarged schematic of Si substrate-PDMS brushes-water molecule layer interface. (B) Front view of the entire system in the second part of the simulation. (C) Schematic of PDMS molecular structure. (D) The water molecule model. (E) The N_2_ molecule model. (F) The O_2_ molecule model. (G) Cross-sectional view of the entire system in the second part of the simulation. (H) The unit cell of single-crystal silicon (100).


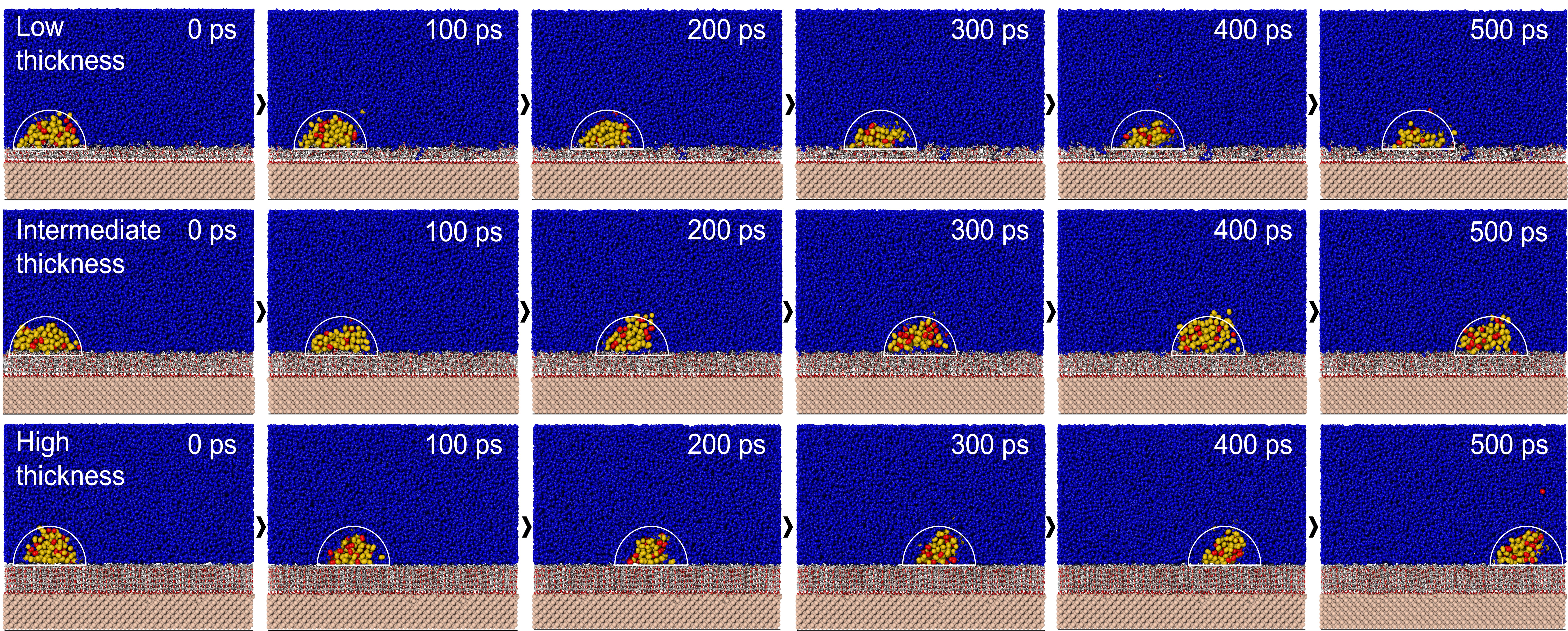


**Figure S21.** Snapshots of bubble sliding on PDMS grafted surfaces of different densities.

**
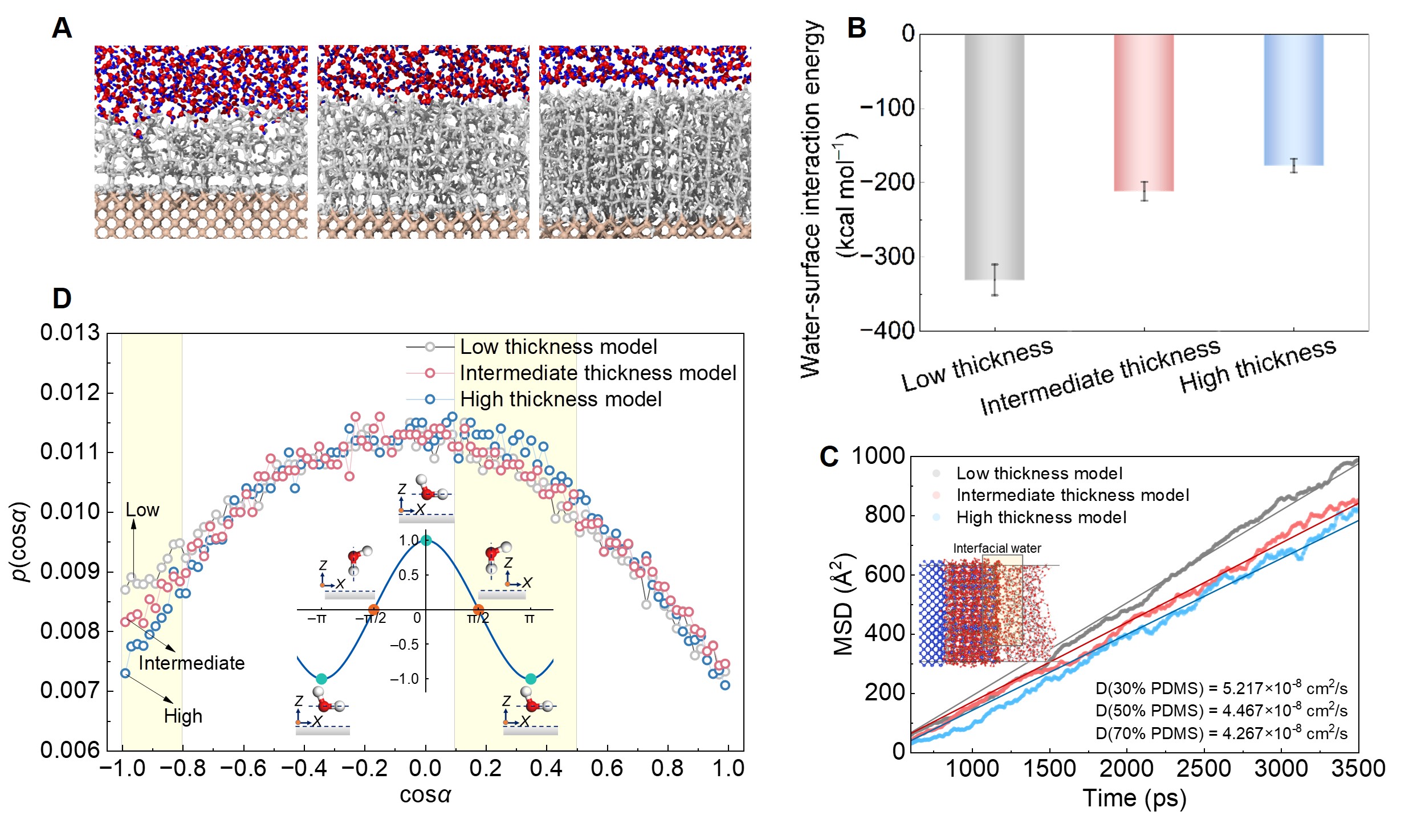
**

**Figure S22.** Quantitative characterization of interfacial water structure at different PDMS brush thicknesses. (A) Side view of the interfacial region from MD simulations showing the effect of brush layer thickness on the arrangement of interfacial water molecules. (B) Time-averaged interaction energies between interfacial water molecules and the CAL surfaces. (C) Mean square displacement (MSD) of interfacial water molecules and the corresponding diffusion coefficients. (D) Orientational distribution of interfacial water molecules near PDMS brushes with different thickness. The orientation is described by cos*α*, where *α* is the angle between the hydroxyl groups vector of a water molecule and the surface normal.

**
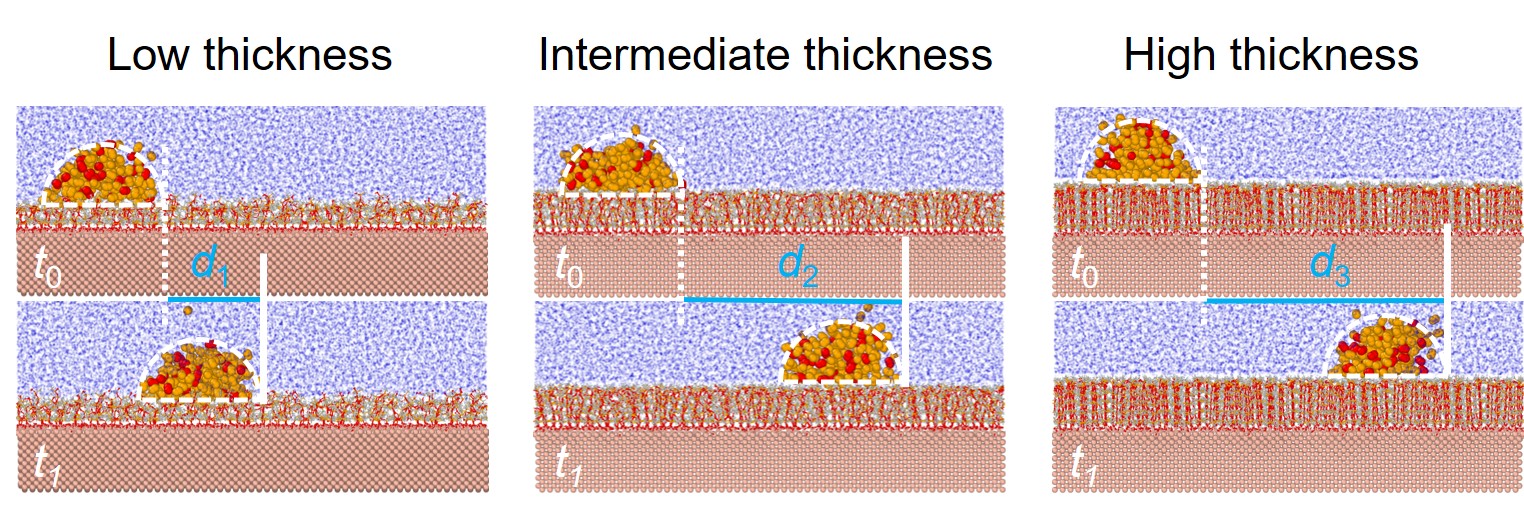
**

**Figure S23.** MD simulations revealing the effect of brush layer thickness on the response to bubble sliding under a constant applied force. The top row shows bubble positions on brush layers at the initial moment (*t*_0_) and after an identical sliding duration (*t*_1_). The resulting sliding distances on brush layers of low (*d*_1_), intermediate (*d*_2_) and high (*d*_3_) thickness increase progressively, indicating an enhancement in bubble velocity.


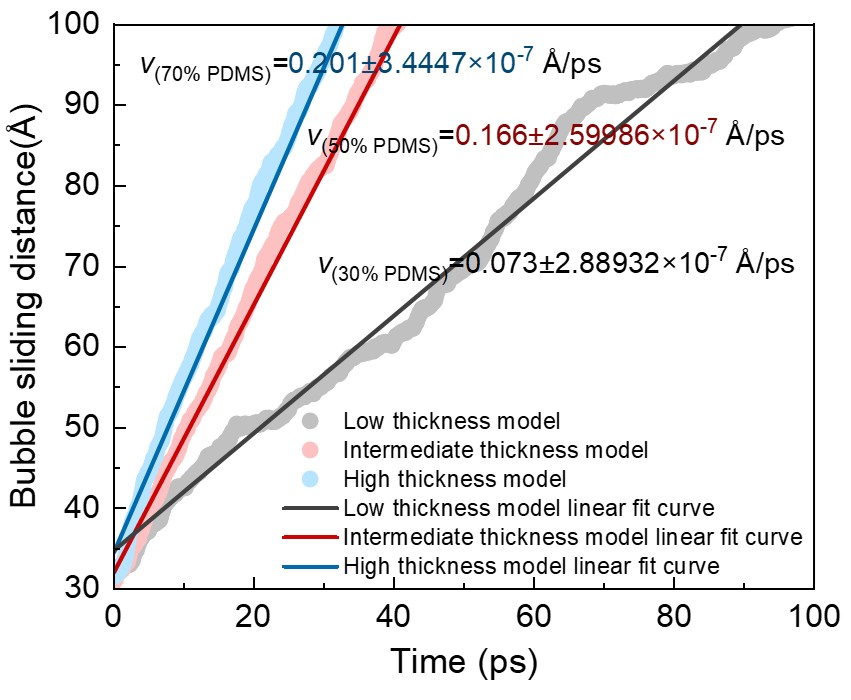


**Figure S24.** Comparison of the time-distance relationship for bubble sliding on PDMS grafted surfaces of different densities. Here, the slope represents the average sliding velocity.

**
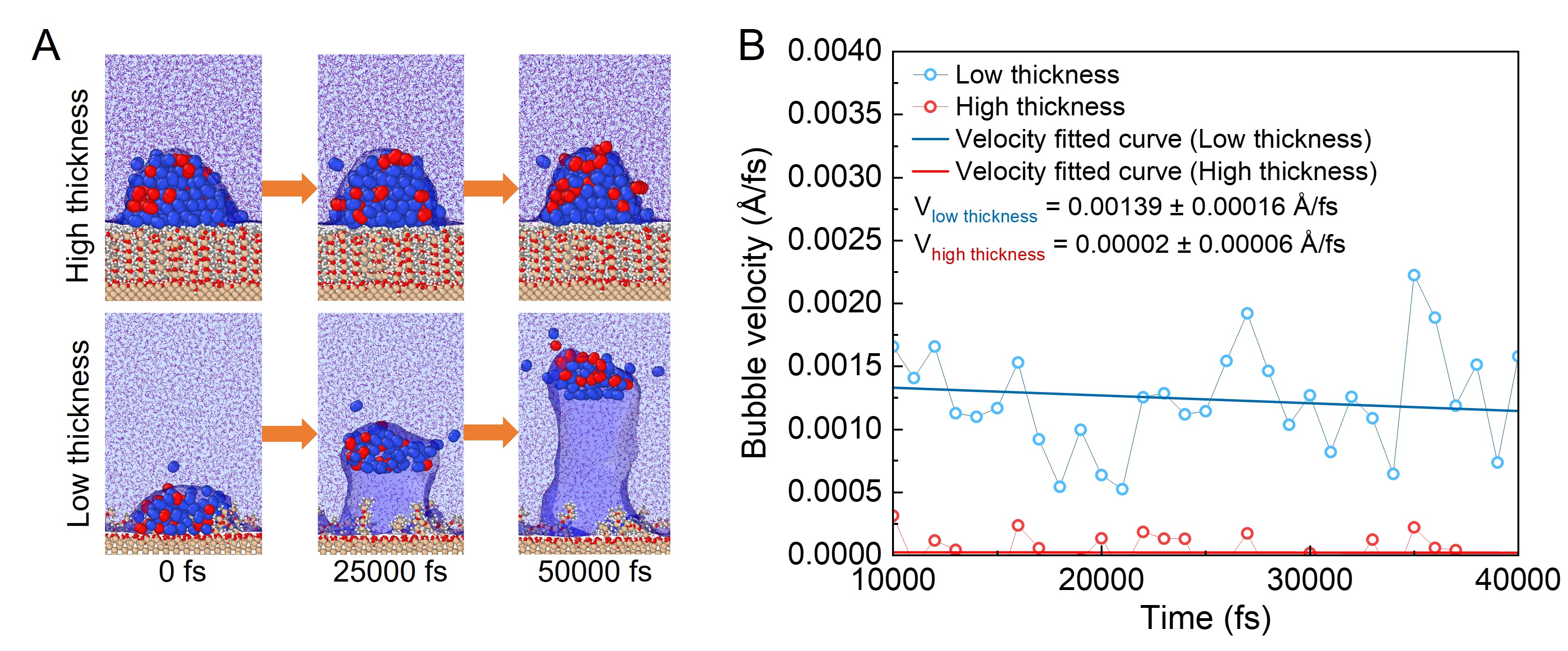
**

**Figure S25.** (A) Representative structural snapshots showing bubble detachment dynamics on PDMS brush layers with low and high thickness under an identical upward driving force. (B) Time-dependent bubble velocity along the z direction during the detachment process.


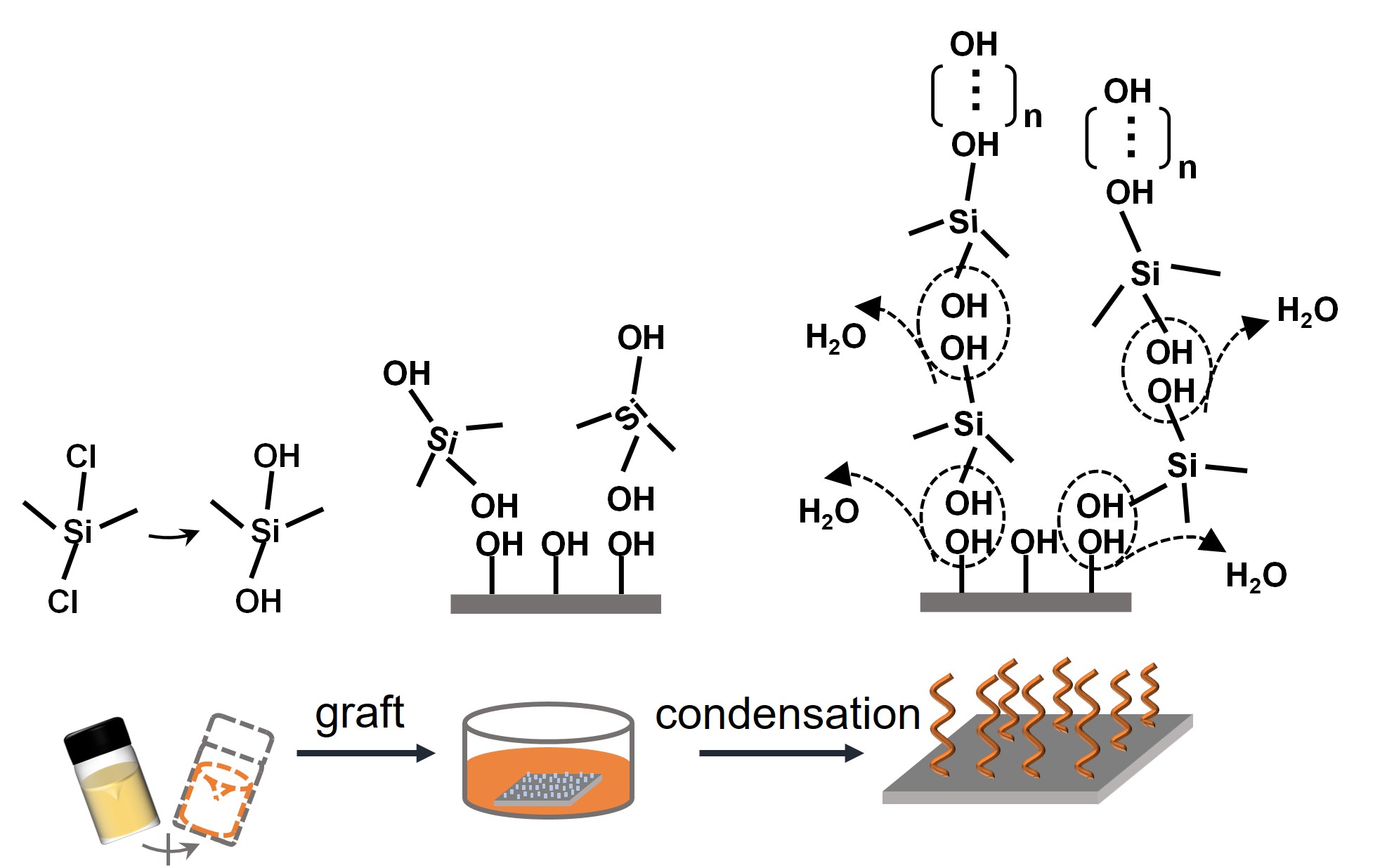


**Figure S26.** Schematic and mechanism of preparing PDMS brushes via the graft and condensation reaction.


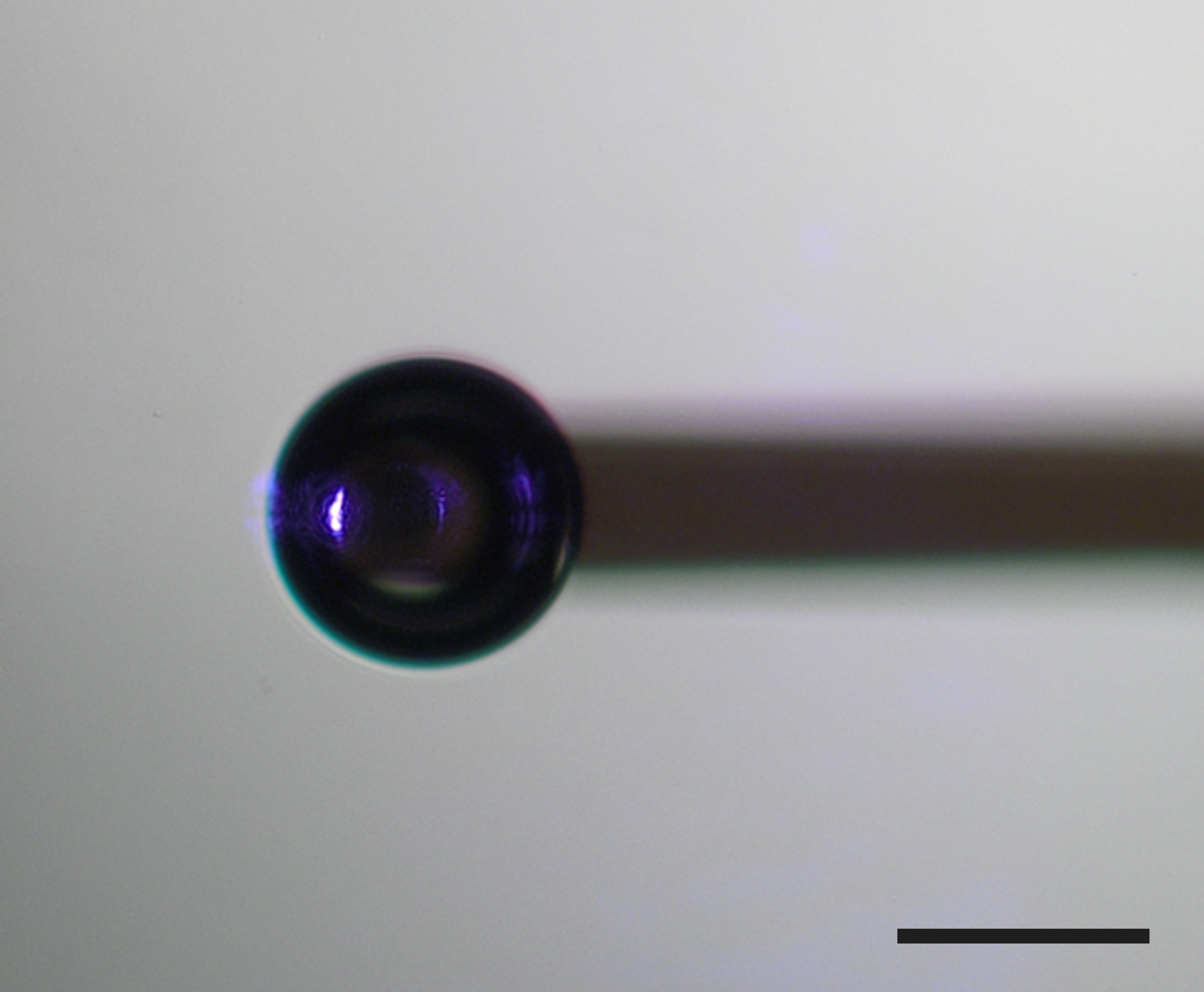


**Figure S27.** Typical microscopic image of AFM bubble probe. Here, a bubble probe with radius *R*_0_ = 62 μm is taken as an example and the scale bar is 100 μm.

Movie S1.

**Attachment tendency experiment of a 1uL bubble onto PDMS network.** The attachment process was displayed to screen using the software accompanying the contact angle measuring instrument and recorded using specific screen recording software.

Movie S2.

**Attachment tendency experiment of a 1uL bubble onto CAL@30.** The attachment process was displayed to screen using the software accompanying the contact angle measuring instrument and recorded using specific screen recording software.

Movie S3.

**Attachment tendency experiment of a 1uL bubble onto CAL@1800.** The attachment process was displayed to screen using the software accompanying the contact angle measuring instrument and recorded using specific screen recording software.

Movie S4.

**MD simulation of bubble sliding on PDMS grafted surface with low thickness.** The movie shows a bubble with 88 air molecules moving on the PDMS surface over a time period of 100 ps.

Movie S5.

**MD simulation of bubble sliding on PDMS grafted surface with intermediate thickness.** The movie shows a bubble with 88 air molecules moving on the PDMS surface over a time period of 100 ps.

Movie S6.

**MD simulation of bubble sliding on PDMS grafted surface with high thickness.** The movie shows a bubble with 88 air molecules moving on the PDMS surface over a time period of 100 ps.

References

[1] A. Kibar, R. Ozbay, M. A. Sarshar, Y. T. Kang, C.-H. Choi, *Langmuir* **2017**, *33*, 12016.

[2] C. Yu, X. Zhu, K. Li, M. Cao, L. Jiang, *Adv. Funct. Mater.* **2017**, *27*, 1701605.

[3] S. Plimpton, *J. Comput. Phys.* **1995**, *117*, 1.

[4] A. P. Thompson, H. M. Aktulga, R. Berger, D. S. Bolintineanu, W. M. Brown, P. S. Crozier, P. J. in ’t Veld, A. Kohlmeyer, S. G. Moore, T. D. Nguyen, R. Shan, M. J. Stevens, J. Tranchida, C. Trott, S. J. Plimpton, *Comput. Phys. Commun.* **2022**, *271*, 108171.

[5] I. J. Gresham, S. G. Lilley, A. R. J. Nelson, K. Koynov, C. Neto, *Angew. Chem. Int. Ed.* **2023**, *62*, e202308008.

[6] B. Khatir, A. Lin, T. V. Vuong, P. Serles, A. Shayesteh, N. S. Y. Hsu, D. Sinton, H. Tran, E. R. Master, T. Filleter, K. Golovin, *Small* **2025**, *21*, 2406089.

[7] J. Lei, D. Huang, W. Zhao, S. Liu, Y. Yue, *Int. J. Heat Mass Transfer* **2024**, *225*, 125407.

[8] H.-P. Fang, J. Hu, *Nucl. Sci. Tech.* **2006**, *17*, 71.

[9] Y.-X. Chen, Y.-L. Chen, T.-H. Yen, *Langmuir* **2018**, *34*, 15360.

[10] S. Maheshwari, M. van der Hoef, X. Zhang, D. Lohse, *Langmuir* **2016**, *32*, 11116.

[11] S. Lepikko, Y. M. Jaques, M. Junaid, M. Backholm, J. Lahtinen, J. Julin, V. Jokinen, T. Sajavaara, M. Sammalkorpi, A. S. Foster, R. H. A. Ras, *Nat. Chem.* **2023**, *16*, 506.

[12] R. W. Hockney, J. W. Eastwood, *Computer Simulation Using Particles*, 0 ed., CRC Press, Boca Raton **2021**.

[13] W. G. Hoover, *Phys. Rev. A* **1985**, *31*, 1695.

[14] P. Dauber-Osguthorpe, V. A. Roberts, D. J. Osguthorpe, J. Wolff, M. Genest, A. T. Hagler, *Proteins: Struct., Funct., Bioinf.* **1988**, *4*, 31.
